# Phthalate Metabolite, Mono(2‐Ethyl‐5‐Hydroxyhexyl) Phthalate (MEHHP), Promotes Uterine‐Fibroid–Associated Phenotypes in Myometrial Stem Cell‐Derived 3D Organoids

**DOI:** 10.1002/tox.70046

**Published:** 2026-02-01

**Authors:** Somayeh Vafaei, Mervat M. Omran, Qiwei Yang, Sandra Madueke‐Laveaux, Ami R. Zota, Ayman Al‐Hendy, Mohamed Ali

**Affiliations:** ^1^ Department of Obstetrics and Gynecology University of Chicago Chicago Illinois USA; ^2^ Oncopathology Research Center, Iran University of Medical Sciences Tehran Iran; ^3^ Cancer Biology Department National Cancer Institute, Cairo University Cairo Egypt; ^4^ Department of Environmental Health Sciences Columbia University Mailman, School of Public Health New York USA; ^5^ Department of Medical Sciences Khalifa University Abu Dhabi UAE; ^6^ Clinical Pharmacy Department, Faculty of Pharmacy Ain Shams University Cairo Egypt

**Keywords:** MEHHP, myometrial stem cells, organoids, phthalates, uterine fibroid

## Abstract

This study investigates how phthalate exposure contributes to uterine fibroid (UF) development by studying the effects of the Mono‐(2‐ethyl‐5‐hydroxyhexyl) phthalate (MEHHP), a metabolite of Di(2‐ethylhexyl) phthalate, on myometrial stem cells (MMSCs). MMSCs from normal (MYON) and at‐risk (MYOF) uterine tissues were cultured in 3D organoids and treated with 1.6 μM MEHHP for 48 h. Functional assays investigated cell viability, apoptosis, and mitochondrial activity, whereas RT‐PCR, immunohistochemistry (IHC), and RNA sequencing evaluated markers of proliferation, apoptosis, extracellular matrix (ECM), and oxidative stress (OS). Cytokines and growth factors secretion were analyzed using a multiplex ELISA. Results showed that MEHHP exposure significantly increased cell viability and inhibited apoptosis in MYOF compared to MYON organoids. Proliferation markers (PCNA, Ki67), anti‐apoptotic markers (BCL2/BAX ratio), and ECM markers (fibronectin and COL1A1) were significantly upregulated, whereas pro‐apoptotic markers (Caspase‐3) were downregulated in MYOF organoids. MEHHP‐treated MYOF organoids exhibited elevated secretion of pro‐inflammatory cytokines (e.g., TNF‐α, IL‐6, IL‐8) and growth factors (e.g., PDGF, VEGF, TGFβ1), indicative of impaired tissue repair and fibrosis. RNA sequencing identified increased OS in MYOF organoids, validated by differential expression of genes such as CA9 and GPX3. Mitochondrial analysis revealed enhanced oxidative phosphorylation (OXPHOS) and elevated oxygen consumption rates, implicating mitochondrial dysfunction as a driver of cytokine release and UF pathogenesis. In conclusion, MEHHP was shown to promote the transformation of MYOF organoids into a UF phenotype by driving proliferation, inhibiting apoptosis, and inducing cytokine‐mediated inflammation via mitochondrial dysfunction. These findings related to MYOF‐specific effects, as compared to MYON, emphasize that these differences are statistically significant and relevant to UF risk. It can shed insight on how phthalates exposures may impact UF pathogenesis and provide a basis for exploring targeted therapeutic strategies.

## 1 Introductions

Uterine fibroids (UFs), (aka; leiomyoma), are prevalent benign tumors in the female pelvis with high incidence, particularly among women of reproductive age and those going through menopause [1]. Unfortunately, the presence of UF is often associated with several symptoms, including heavy menstrual bleeding (HMB), pelvic pressure or pain, fatigue, abdominal distention, and frequent urination [2, 3, 4, 5]. Furthermore, UF can contribute to complications such as anemia, dysmenorrhea, backache or leg pains, recurrent miscarriage, and infertility [6]. Given the substantial impact of UF on women's daily lives, it is critical to consider various associated risk factors. These include early menarche, nulliparity, age, African American descent, vitamin D deficiency, obesity, positive family history of UF, and exposure to endocrine‐disrupting chemicals (EDCs) [7, 8, 9, 10, 11, 12, 13, 14, 15, 16]. EDCs, via acting as hormone agonists or antagonists, can change hormone activities in the body following exposure to different sources such as personal care products (e.g., cosmetics), diet, medications, as well as contact with toys and plastics [17]. Although the etiology of UF is still not fully understood, some prior studies have linked exposures to EDCs, such as phthalates, to increased UF risk [18, 19]. Among the group of phthalate chemicals, di‐(2‐ethylhexyl)‐phthalate (DEHP) has most consistently been implicated with adverse fibroid outcomes [20, 21, 22]. One of the primary metabolites of DEHP is mono‐(2‐ethyl‐5‐hexyl) phthalate (MEHHP) [23]. DEHP undergoes hydrolysis to mono(2‐ethylhexyl) phthalate (MEHP), followed by cytochrome P450‐mediated oxidation into secondary metabolites including MEHHP. These metabolites are excreted in urine as glucuronide conjugates, with MEHHP being one of the major oxidative products detected in human populations. NHANES biomonitoring data (1999–2016) shows that MEHHP was detectable in over 95% of urine samples from U.S. women aged 16–49 and children aged 6–17, with median concentrations ranging from 13.7–28.9 ng/mL across survey cycles, reflecting widespread exposure to DEHP [24, 25]. However, concentrations declined over time, likely due to regulatory changes in phthalate use, as highlighted by Zota et al. who observed a 37% decrease in ΣDEHP metabolites between 2001 and 2010 in the general U.S. population [26].

Recent studies, including work by Serdar Bulun, have highlighted the mechanisms by which phthalates, particularly DEHP and its metabolite MEHHP, promote UF growth. MEHHP activates the aryl hydrocarbon receptor (AHR), increasing tryptophan uptake and kynurenine production, which drives fibroid cell survival and proliferation while reducing apoptosis. These effects are most pronounced in fibroid cells with *MED12* mutations. Epidemiological evidence also links higher urinary MEHHP levels to increased UF risk. However, these studies focus exclusively on UF pathology and do not explore the effects of phthalates on normal myometrial tissue as a potential pre‐UF stage.

Like other diseases, the “multiple hit” concept has been implicated in the etiology of UF. Our prior research suggests that UF originates from perturbed MMSCs, with the “first hit” triggered by early‐life, in utero exposure to EDCs. These disrupted MMSCs, when exposed to a “second hit” during adolescence, may develop genomic instability with subsequent mutations such as the one in the *MED12* gene, driving the development of nearly 80% of fibroid tumors. The second hit can be initiated through various risk factors, with exposure to EDCs as a significant factor. Notably, both our work and others have shown that early‐life EDC exposures elevate the risk of UF in Eker rats, the sole authentic animal model of UF, carrying a germline TSC2 mutation. Intriguingly, *MED12* and TSC2 defects share common downstream signaling pathways, involving the activation of DNA damage, estrogen signaling, and pro‐inflammatory signaling [27]. Crucially, to date, no experimental studies have fully examined the role of phthalate exposure, either as early life, in the early stages of fibroid development [28, 29].

We were the first to report the development of a 3D organoid model for human MYO and UF tissues, derived from their corresponding stem cells, in vitro. This model retains essential aspects of tissue scaffolding, enabling MMSCs to undergo appropriate differentiation and proliferation in response to estrogen and thus mimicking the in vivo microenvironment [30, 31, 32]. This is a notable feature often lost when cells are grown in 2D monolayers. Organoids are three‐dimensional (3D) structures that resemble miniaturized organs, consisting of self‐organized cells derived from stem cells or tissue samples [30, 33]. Our patient tissue‐derived stem cell organoids are helpful for advancing UF related research and clinical applications due to their ability to accurately replicate myometrial and UF phenotypes [34].

Intriguingly, an increasing body of evidence suggests the potential involvement of oxidative stress (OS) in the development of UF [35, 36]. OS, characterized by an imbalance between prooxidants and antioxidants, manifests through complex cascades involving angiogenesis, hypoxia, dietary factors, and environmental exposures [37, 38, 39]. This imbalance leads to cellular damage and activates inflammatory pathways, thereby fostering UF growth. Furthermore, emerging evidence points to phthalates' potential to induce OS, as studies reveal associations between phthalate metabolites and increased lipid peroxidation, inflammation, and reduced antioxidant levels [40]. Mitochondria play a critical role in cellular energy production through oxidative phosphorylation (OXPHOS), which generates ATP [41]. However, this process also produces reactive oxygen species (ROS), and an imbalance in ROS levels can lead to OS and cellular damage [42]. In the context of UF, increased ROS levels have been shown to contribute to DNA damage and activating several signaling pathways that promote UF growth [43]. Additionally, fibroids may have impaired antioxidant defense systems, exacerbating OS [35, 44]. This ongoing study is exploring the effects of MEHHP metabolites and its potential impacts towards UF phenotype using a 3D organoid system. Therefore, this study aimed to investigate the effects of MEHHP exposure, on human tissue derived Myometrial Stem Cells (MMSCs) at risk of developing UF, since isolated from the UF‐containing uterus (named MYOF), and compare it to normal MMSCs, isolated from fibroid free uterus (named MYON) following MEHHP exposure, in a 3D organoid culture system.

## 2 Materials and Methods

### 2.1 Isolation and Purification of MYOF and MYON Stem Cells

To create a 3D in vitro model that accurately replicates the complexity of in vivo conditions, we isolated MMSCs from patient samples using Stro‐1/CD44 surface markers and FACS as previously described [31]. These markers specifically enrich a subpopulation of myometrial cells that possess important characteristics of stem/initiating/progenitor cells. We collected myometrial samples of MYOF and MYON from women between the ages of 30 and 50 who were undergoing hysterectomy for symptomatic UF (for MYOF) or other reasons such as vaginal prolapse (for MYON). All human tissue samples used in this study were carefully matched by age, BMI, and menstrual cycle phase, consistent with our previously published work [30, 31, 32]. The selection process excluded individuals with other gynecological malignancies. The MMSCs were maintained using a combination of DMEM/F12 medium supplemented with 12% Fetal Bovine Serum (FBS) from Omega Scientific (Fisher Scientific) and expanded in flasks coated with Attachment Factor Protein from GibcoTM (Fisher Scientific) under hypoxia (2% O_2_ and 5% CO_2_) culture condition in a 37°C incubator.

### 2.2 Organoid 3D Formation and Exposure to MEHHP

Upon reaching confluence, the MYOF and MYON MMSCs were dissociated using a solution of 0.25% Trypsin and 0.1% EDTA in HBSS without calcium or magnesium (Fisher Scientific, Waltham, MA). The resulting cell pellet was collected through centrifugation, and the supernatant was carefully discarded. The cell pellet was then resuspended in serum‐free MesenCult‐ACF Plus Medium (Stem cell Technologies, Vancouver, Canada); any clumps were disrupted by gentle pipetting and kept on ice. Cell counting was conducted using a TC20 automated cell counter (Bio‐Rad Laboratories, Hercules, CA). Around 10 000 cells were combined with 2 μL of Matrigel (Corning, Corning, NY) in a final volume of 100 μL per well. The cell mixture was subsequently added to V‐bottom plates, specifically Akura 96 Spheroid Microplates (InSphero), followed by centrifugation. The plates were then placed in a 37°C incubator with 5% CO_2_ for 30 min. Afterward, an additional 100 μL was added to each well [30]. Following a 7‐day incubation period in normoxia (20% O_2_ and 5% CO_2_) culture condition (Figure S1A), the organoids were washed twice with Dulbecco's Phosphate‐Buffered Saline (DPBS). Subsequently, they were exposed to two different treatments: MEHHP (Toronto Research Chemicals, Canada Cat. No. M542510) at a concentration of 1.6 μM and sterile filtered dimethyl sulfoxide (DMSO) (Tocris Bioscience, Canada Cat. No. 3176), which was used as Vehicle (VEH) to dissolve MEHHP and served as the untreated control. We selected 1.6 μM MEHHP as an environmentally relevant, intermediate concentration within the effective in vitro range reported to enhance uterine fibroid cell survival and reduce apoptosis, rather than a supraphysiologic toxic dose. Consistent with prior work on MEHHP‐mediated activation of pro‐survival pathways in leiomyoma cells [23], a 48‐h exposure was used as an acute, short‐term treatment window that is sufficient to elicit downstream transcriptional and phenotypic changes in myometrial organoids towards a fibroid‐like state. In addition, large biomonitoring studies (NHANES and longitudinal pregnancy cohorts) demonstrate that oxidized DEHP metabolites such as MEHHP and MEOHP are widely detectable and often predominate over MEHP, with relatively stable urinary levels over time, supporting that our low‐micromolar, short‐term in vitro exposure models environmentally relevant, recurrent human exposure [45, 46]. Treatments were prepared in DMEM/F12 phenol red‐free media supplemented with Insulin‐Transferrin‐Selenium (ITS) (Gibco) and Human Recombinant Epidermal Growth Factor (Millipore). Organoids in this study were generated from three MYON donors and four MYOF donors. Data shown in the results represents three independent experiments; each performed in triplicate.

### 2.3 Assessment of Viability and Apoptosis

Viability assessment was conducted in four experimental groups: MYOF‐VEH, MYOF‐MEHHP, MYON‐VEH, and MYON‐MEHHP using the CellTiter‐Glo 3D Cell Viability Assay (Promega, Germany), specifically designed for determining cell viability in 3D organoids. This assay utilizes ATP as an indicator of viability and generates a highly sensitive luminescence readout, surpassing the capabilities of colorimetric or fluorescence‐based methods in culture medium. To initiate the assay, an equivalent volume of CellTiter‐Glo 3D Reagent to the volume of the cell culture medium present in each well was added. The contents were vigorously mixed for 5 min to induce cell lysis. Subsequently, the plate was incubated at room temperature for an additional 25 min. This stabilized luminescence signal was then recorded as a measure of viability.

Similarly, RealTime‐Glo Annexin V Apoptosis and Necrosis Assay (Promega, Germany) was performed for all same four experimental groups. This assay is designed to provide real‐time and kinetic measurements of apoptosis and necrosis in live cells without cell lysis. It specifically detects the exposure of phosphatidylserine (PS) on the outer leaflet of the cell membrane, which is a well‐established and validated indicator of apoptosis. The assay utilizes annexin V binding to detect PS exposure, and this binding event is detected using a luminescence signal. Additionally, necrosis is detected using a fluorescence signal (485 nm_Ex_/520–30 nm_Em_). This combination of signals allows for the reliable assessment of apoptosis and necrosis in a dynamic and time‐dependent MEHHP manner.

### 2.4 Organoid Block Generation and Immunohistochemistry Staining

The four groups of treated organoids were fixed and sent to the Organoid and Primary Culture Research Core at the University of Chicago for preparing paraffin‐embedded organoid blocks. Subsequently, staining with Hematoxylin and Eosin (H&E) as well as Trichrome staining was conducted to visualize the tissue structure and ECM deposition. Immunohistochemical (IHC) staining was performed at the University of Chicago Pathology Core Facility. Proliferation markers, including *PCNA* and *Ki67*, were investigated to assess cellular proliferation within the organoids [47]. Antibody information is summarized in the provided Table 1. Additionally, apoptotic markers, such as *BAX*, *BCL2*, and *Caspase3*, were examined to evaluate apoptotic processes within the organoids [48, 49]. The expression levels of Fibronectin (*FN*) and *COL1A1* were also analyzed to assess the composition of the ECM [50, 51]. Moreover, the expression of 8‐hydroxy‐2′‐deoxyguanosine *(8‐OHdG)* and phosphorylated histone *H2AX* (*γH2AX*) was investigated [52]. *8‐OHdG* serves as a marker for oxidative DNA damage, specifically guanine residue modification [53]. *γH2AX*, on the other hand, is a hallmark marker for DNA double‐strand breaks, which can be induced by various factors, including OS [28]. The slides were scanned and analyzed using the Aperio ImageScope—Pathology Slide Viewing Software. Additionally, a semi‐quantitative analysis was conducted, and the slides were scored using the H‐score method by an independent pathologist. The H‐score considers both the percentage of stained cells and the intensity of staining, resulting in a score ranging from 0 to 300. Staining intensity is categorized as follows: “0” for absent expression, “1” for weak staining, “2” for moderate staining, and “3” for strong staining. The H‐score calculation incorporates these categories as follows: (1 × percentage of cells with weak staining) + (2 × percentage of cells with moderate staining) + (3 × percentage of cells with strong staining). This scoring method provides a comprehensive evaluation of staining patterns, considering both the extent and intensity of staining within the tissue samples. It allows for a more objective and standardized assessment of the expression levels of the investigated markers [54].

**TABLE 1:** List of primary antibodies used in experiments.

Antibodies names	Optimized dilution	Optimized dilution	Incubation time
Anti‐PCNA antibody [PC10] (ab29)	PCNA	1:1000	25 m
Anti‐Bax antibody [E63] (ab32503)	BAX	1:800	1 h
Anti‐Bcl‐2 antibody (ab59348)	BCL2	1:250	1 h
Cleaved Caspase‐3 (Asp175) Antibody #9661	CASPASE3	1:200	1 h
Anti‐Fibronectin antibody (ab2413)	Fibronectin	1:400	1 h
COL1A1 (E8F4L) XP Rabbit mAb #72026	COL1A1	1:400	1 h
8‐OHdG (E‐8) sc‐393 871	8‐OHdG	1:50	1 h
γH2AX (Ser139) (20E3) Rabbit mAb #9718	γH2AX	1:50	1 h

### 2.5 Organoid RNA Sequencing

RNA sequencing (RNA‐seq) analysis was performed on four experimental groups (MYOF‐VEH, MYOF‐MEHHP, MYON‐VEH, and MYON‐MEHHP in triplicate). First, the quality of the samples was assessed and reported by Novogene Company. Total RNA was used to purify messenger RNA (mRNA) using poly‐T oligo‐attached magnetic beads. Subsequently, a library was constructed, and the first strand of cDNA was synthesized using random hexamer primers, followed by the synthesis of the second strand of cDNA. The library was then checked for quantification using Qubit and RT‐PCR, and for size distribution detection using a bioanalyzer. The quantified libraries were pooled and sequenced on Illumina platforms, based on the effective library concentration, and desired data amount. The index‐coded samples were clustered according to the manufacturer's instructions, and the library preparations were sequenced on an Illumina platform, generating paired‐end reads [55]. Raw data in fastq format underwent quality control and cleaning. Clean reads were aligned to a reference genome using Hisat2, and gene counts were determined with Feature Counts. FPKM values were calculated for gene expression levels. Differential expression analysis using DESeq2 identified genes with adjusted *p* values ≤ 0.05 as differentially expressed [56].

Gene Set Enrichment Analysis (GSEA) was performed to evaluate whether predefined gene sets exhibit consistent and significant differences between two biological states. Genes were ranked based on the degree of differential expression in the samples [57]. To analyze the functional characteristics of the differentially expressed genes (DEGs), Gene Ontology (GO) enrichment analysis was performed using the clusterProfiler R package, with correction for gene length bias [58]. GO terms with a corrected *p* value less than 0.05 were considered significantly enriched by the differentially expressed genes. Additionally, the clusterProfiler R package was used to assess the statistical enrichment of DEGs in KEGG pathways, which provide insights into high‐level functions and utilities of biological systems [59]. Reactome pathways, which encompass reactions and biological pathways in human model species, were also analyzed for enrichment [60]. The DO (Disease Ontology) database, which describes the relationship between human genes and diseases, was used to identify significantly enriched DO pathways [61]. Furthermore, the DisGeNET database, integrating information on human disease‐related genes, was utilized to identify significantly enriched DisGeNET pathways [62]. The statistical enrichment of differentially expressed genes in Reactome, DO, and DisGeNET pathways was determined using the clusterProfiler software [63].

### 2.6 Organoid RNA Isolation and qRT‐ PCR

For RNA extraction, the miRNeasy Tissue/Cells Advanced Micro Kit Cat. No. 217684 (Qiagen, Valencia, CA) was employed according to the manufacturer's instructions. Reverse transcription was conducted using the RNA to cDNA EcoDry Premix Kit (Takara Bio USA, Cat. No. 639549). RT‐PCR was performed on the CFX Connect RT‐PCR Detection System (Bio‐Rad Laboratories, Hercules, CA) using the Advanced Universal SYBR Green qPCR Mastermix (TaKaRa, Tokyo, Japan). The organoids were subjected to gene expression examination of *PCNA*, *Ki67*, *BAX, BCL2*, and *Caspase3*. *FN1* and *COL1A1*. Moreover, based on the RNA‐Seq results, we validated the expression levels of top dysregulated genes involved in various cellular processes including Carbonic anhydrase 9 (*CA9*), which plays a vital role in OS responses. Cytochrome C Oxidase Subunit 6C (*COX6C*), a gene associated with cellular respiration and Glutathione peroxidase (*GPX3*); a gene known for its significant role in antioxidant defense mechanisms. Conversely, we measured the expression of Paired Box 8 (*PAX8*), a transcription factor that participates in various cellular processes. Moreover, we included the transcription factor nuclear factor erythroid 2‐like (*NFE2L*), which plays a crucial role in cellular responses to OS, in our analysis. Furthermore, we quantified the expression of the Xeroderma Pigmentosum Group A‐Complementing Protein (*XPA*) gene to gain insights into the DNA repair mechanisms within the organoids. The PCR primers sequences used in the experiments can be found in the provided Table 2, and the expression levels of all measured genes were normalized to *GAPDH*. The PCR amplification procedure included an initial pre‐denaturation step at 95°C for 30 s, followed by 40 cycles of denaturation at 95°C for 15 s, annealing at 60°C for 30 s, and extension at 65°C for 31 s. Since a balanced mitochondrial DNA copy number (mtDNAcn) is essential for maintaining optimal mitochondrial function, as insufficient or excessive mtDNAcn can lead to mitochondrial dysfunction and impaired energy production. Therefore, mtDNAcn has emerged as a reliable marker that reflects mitochondrial function and the response to OS [64]. To determine the relative normalized amount of mtDNAcn, a ratio of mtDNA to nuclear DNA (nDNA) was calculated. RT‐PCR reactions were conducted using the StepOne RT‐PCR system as previously described [65]. The specific primer sequences for human samples were reported in the Table 2. The PCR amplification procedures were as follows: pre‐denaturation at 95°C for 5 min, followed by 40 cycles of (94°C for 30 s, 58°C for 30 s, 72°C for 60 s) [66]. The relative expression of the target gene was calculated using the 2^−ΔΔCT^ method [67]. All qPCR data are presented as the mean ± SEM (standard error of the mean) of biological triplicates. Statistical analysis was performed using an unpaired *t*‐test, and *p* values less than 0.05 were considered statistically significant in a two‐tailed analysis [68].

**TABLE 2:** List of genes and primers used for qRT‐PCR.

Symbol	Forward primer sequence (5′‐3′)	Reverse primer sequence (5′‐3′)
GAPDH	GTCTCCTCTGACTTCAACAGCG	ACCACCCTGTTGCTGTAGCCAA
PCNA	CAAGTAATGTCGATAAAGAGGAGG	GTGTCACCGTTGAAGAGAGTGG
Ki67	GAAAGAGTGGCAACCTGCCTTC	GCACCAAGTTTTACTACATCTGCC
BCL2	ATCGCCCTGTGGATGACTGAGT	GCCAGGAGAAATCAAACAGAGGC
BAX	ATG TTT TCT GAC GGC AAC TTC	AGT CCA ATG TCC AGC CCA T
Caspase 3	GGAAGCGAATCAATGGACTCTGG	GCATCGACATCTGTACCAGACC
TGFB1	TACCTGAACCCGTGTTGCTCTC	GTTGCTGAGGTATCGCCAGGAA
Fibronectin	ACAACACCGAGGTGACTGAGAC	GGACACAACGATGCTTCCTGAG
COL1A1	GATTCCCTGGACCTAAAGGTGC	AGCCTCTCCATCTTTGCCAGCA
CA9	GTGCCTATGAGCAGTTGCTGTC	AAGTAGCGGCTGAAGTCAGAGG
GPX3	TACGGAGCCCTCACCATTGATG	CAGACCGAATGGTGCAAGCTCT
COX6C	GTAGCATTCGTGCTATCCCTGG	GATACCAGCCTTCCTCATCTCC
PAX8	TCAACCTCCCTATGGACAGCTG	GAGCCCATTGATGGAGTAGGTG
NFE2L	CACATCCAGTCAGAAACCAGTGG	GGAATGTCTGCGCCAAAAGCTG
XPA	GAAGTCCGACAGGAAAACCGAG	GATGAACAATCGTCTCCCTTTTCC
mtDNA	CACCCAAGAACAGGGTTTGT	TGGCCATGGGTATGTTGTTAA
nDNA	TAGAGGGACAAGTGGCGTTC	CGCTGAGCCAGTCAGTGT

### 2.7 Multiplex Cytokines Array

The Human Cytokine/Chemokine Panel A 48‐Plex Discovery Assay Array (HD48A) was performed using the Luminex 200 system (Luminex, Austin, TX, USA) by Eve Technologies Corp. (Calgary, Alberta) to test the samples and generate results for a wide range of significant biomarkers. Through the analysis of these biomarkers, valuable information about the cellular and molecular characteristics of the MEHHP‐exposed MYOF and MEHHP‐exposed MYON organoids can be obtained, shedding light on the cytokine and chemokine profiles present in the samples. The 48‐plex consisted of Solute Carrier Family 40 Member Ligand (sCD40L), Epidermal Growth Factor (EGF), Eotaxin, Fibroblast Growth Factor‐2 (FGF‐2), FMS‐Like Tyrosine Kinase 3 Ligand (FLT‐3 Ligand), Fractalkine, Granulocyte Colony‐Stimulating Factor (G‐CSF), Granulocyte‐Macrophage Colony‐Stimulating Factor (GM‐CSF), Growth‐Regulated Oncogene Alpha (GROα), Interferon Alpha‐2 (IFN‐α2), Interferon Gamma (IFN‐γ), Interleukin‐1 Alpha (IL‐1α), Interleukin‐1 Beta (IL‐1β), Interleukin‐1 Receptor Antagonist (IL‐1RA), Interleukin‐2 (IL‐2), Interleukin‐3 (IL‐3), Interleukin‐4 (IL‐4), Interleukin‐5 (IL‐5), Interleukin‐6 (IL‐6), Interleukin‐7 (IL‐7), Interleukin‐8 (IL‐8), Interleukin‐9 (IL‐9), Interleukin‐10 (IL‐10), Interleukin‐12 Subunit p40 (IL‐12(p40)), Interleukin‐12 Subunit p70 (IL‐12(p70)), Interleukin‐13 (IL‐13), Interleukin‐15 (IL‐15), Interleukin‐17A (IL‐17A), Interleukin‐17E/Interleukin‐25 (IL‐17E/IL‐25), Interleukin‐17F (IL‐17F), Interleukin‐18 (IL‐18), Interleukin‐22 (IL‐22), Interleukin‐27 (IL‐27), Interferon Gamma‐Inducible Protein‐10 (IP‐10), Monocyte Chemoattractant Protein‐1 (MCP‐1), Monocyte Chemoattractant Protein‐3 (MCP‐3), Macrophage Colony‐Stimulating Factor (M‐CSF), Macrophage‐Derived Chemokine (MDC), Monokine Induced by Gamma Interferon (MIG)/C‐X‐C Motif Chemokine Ligand 9 (CXCL9), Macrophage Inflammatory Protein‐1 Alpha (MIP‐1α), Macrophage Inflammatory Protein‐1 Beta (MIP‐1β), Platelet‐Derived Growth Factor‐AA (PDGF‐AA), Platelet‐Derived Growth Factor‐AB/BB (PDGF‐AB/BB), Regulated upon Activation, Normal T Cell Expressed and Presumably Secreted (RANTES), Transforming Growth Factor Alpha (TGFα), Tumor Necrosis Factor Alpha (TNF‐α), Tumor Necrosis Factor Beta (TNF‐β), Vascular Endothelial Growth Factor A (VEGF‐A). Individual analyte sensitivity values are available in the MILLIPLEX MAP protocol.

### 2.8 ELISA Assay

The enzyme‐linked immunosorbent assay (ELISA) analysis was employed using two specific kits: the 8‐OHdG ELISA Kit (Abcam ab201734) and 8‐iso‐prostaglandin F2 alpha (8‐iso‐PGF2 alpha) ELISA Kit (Abcam ab133025). To perform the assay, samples or standards are added to the microplate wells, facilitating the binding of 8‐OHdG or 8‐iso‐PGF2α to the immobilized antibodies. Following a washing step to remove unbound materials, an enzyme‐linked secondary antibody is added, then a substrate is subsequently added, generating a colorimetric signal that is directly proportional to the quantity of 8‐OHdG or 8‐iso‐PGF2α in the samples. The absorbance is measured using a microplate reader, enabling the determination of 8‐OHdG concentrations by comparing them to the standard curve.

### 2.9 Mitochondria Functional Assay

We assessed mitochondrial function using the Agilent Technologies XF Cell Mito Stress Test Kit (Cat. No. 103010‐100) to determine cellular oxygen consumption rate (OCR), which facilitated detailed investigations of cellular respiration and energy metabolism. Cells were incubated in XF base medium in an ambient air incubator for 1 h before starting the assay. The samples were mixed for 3 min and measured for 3 min using an XFe96 Extracellular Flux Analyzer (Seahorse Bioscience). Oligomycin (2.5 μM), carbonyl cyanide‐4 (trifluoromethoxy) phenylhydrazone (FCCP) (2 μM), and rotenone/antimycin A (0.5 μM) were injected at the indicated time points. Finally, the OCR data were normalized to account for the differences in the initial cell numbers for each well. The mitochondria stress test protocol provides information on basal respiration, ATP‐linked respiration, proton leak, maximal respiration capacity, and non‐mitochondrial respiration of cells [69]. Therefore, this assay can be used to provide insight on the mechanism of action of compounds that directly target mitochondrial respiration [70].

### 2.10 Statistical Analysis

All raw data was collated in a Microsoft Excel database, whilst SPSS 18.0 was used for statistical analysis. The data results are presented in two ways as mean ± SD or mean ± SE respectively. One‐way and two‐way ANOVA was used to carry out analysis of variance between the groups, with significant differences accepted at *p* < 0.05 and the extremely significant difference acceptance at *p* < 0.01 confidence levels, respectively.

## 3 Results

### 3.1 MEHHP Exposure Results in Exaggerated Increase in Cell Viability and Apoptosis Impairment in At‐Risk MYOF Organoids

Fresh myometrial tissues were utilized to isolate MMSCs (MYOF *n* = 4 and MYON *n* = 3), and then 3D organoids were developed and allowed to mature for 7 days (Figure S1A). Subsequently, the organoids were subjected to either 1.6 μM of MEHHP or VEH for 48 h.

**FIGURE 1:**
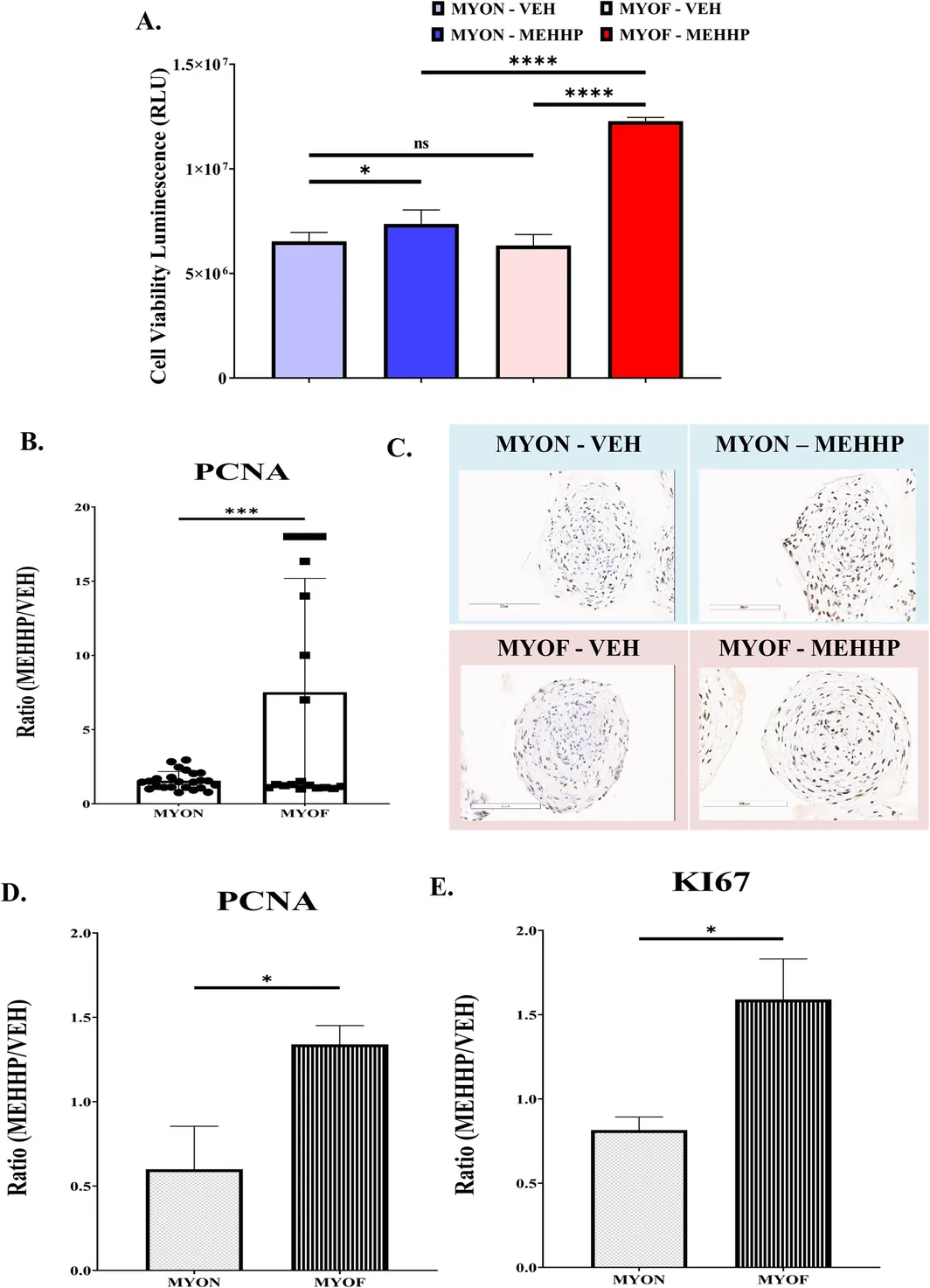
3D organoid MEHHP exposure leads to increased cell viability. (A) The cell Titer‐Glo 3D cell viability assay reveals an augmented viability in MEHHP‐exposed MYOF organoids, indicating enhanced cellular survival and function via ELISA. (B, C) Compared to MEHHP‐exposed MYON organoids, MEHHP‐exposed MYOF organoids exhibit an elevated expression of proliferating cell nuclear antigen (PCNA), indicating an intensified behavioral response via IHC analysis. (D) MEHHP‐exposed MYOF organoids induce a significant upregulation of PCNA expression, indicating enhanced cellular proliferation via RT‐PCR analysis. (E) MEHHP‐exposed MYOF organoids lead to a substantial elevation in KI67 expression, suggesting a pronounced increase in cellular proliferation via RT‐PCR analysis. Error bars represent the mean + SD. *p < 0.05; **p < 0.01; ***p < 0.001. No significant difference. *p* values were analyzed by unpaired two‐tailed Student's *t*‐test and two‐way ANOVA.

Viability assay exhibited a notable increase in MEHHP exposed MYOF organoid viability for 48 h, compared to the untreated control, VEH exposed MYOF organoid (*p* < 0.001), unlike MEHHP exposed MYON organoids (Figure 1A). Furthermore, analysis of PCNA protein expression using the IHC demonstrated a significant increase in the MEHHP‐exposed groups with MYOF (MEHHP/VEH ratio) showing exaggerated increase compared to MYON (MEHHP/VEH ratio) (*p* < 0.001) (Figure 1B,C). Consistent with these findings, RT‐PCR analysis revealed elevated levels of *PCNA* and *KI67* gene expression (Figure 1D,E) in response to MEHHP exposure in MYOF compared to MYON organoids. The observed increase in PCNA protein expression and upregulated gene expression of *PCNA* and *Ki67* further suggests a possible involvement of MEHHP in stimulating UF cellular growth especially in at‐risk MYOF. These results point towards a potential effect of MEHHP exposure to MYOF derived organoid in promoting cell proliferation and survival.

**FIGURE 2:**
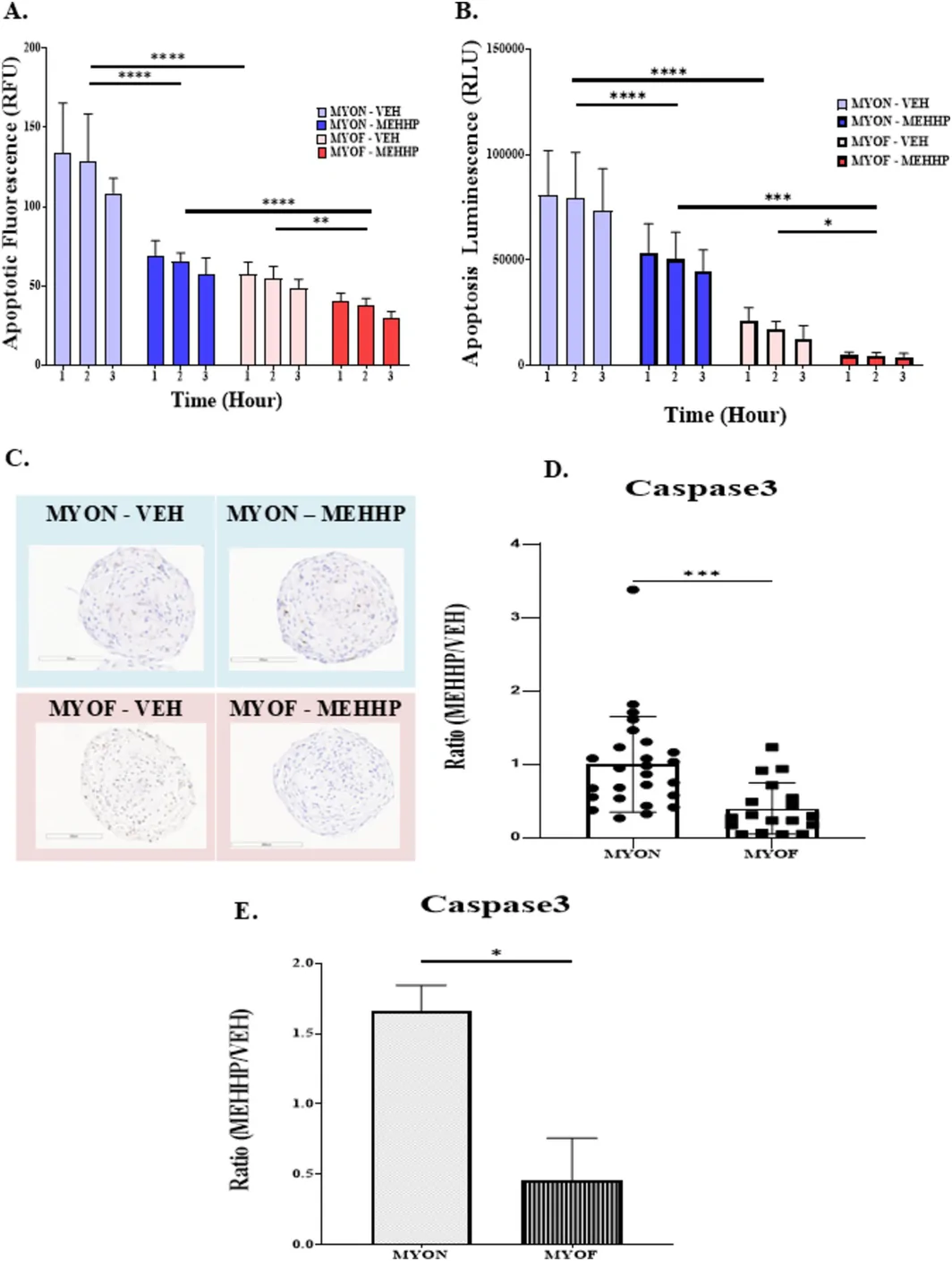
3D organoid MEHHP exposure leads to decreased apoptosis. (A, B) Apoptosis functional assay. The RealTime‐Glo Annexin V Apoptosis and Necrosis Assay reveals a decrease in apoptosis fluorescence and luminescence in MEHHP‐exposed MYOF organoids, suggesting a reduction in programmed cell death. (C, D) MEHHP‐exposed MYOF organoids lead to a notable decrease in CASPASE3 expression, suggesting a reduction in apoptotic activity and indicating a potential suppression of programmed cell death via IHC analysis. (E) MEHHP‐exposed MYOF organoids lead to a notable decrease in CASPASE3 expression, suggesting a downregulation of apoptotic activity and indicating a potential suppression of programmed cell death pathways via RT‐PCR analysis. Error bars represent the mean + s.d. *p < 0.05; **p < 0.01; ***p < 0.001. n.s., no significant difference. *p* values were analyzed by unpaired two‐tailed Student's *t*‐test and two‐way ANOVA.

Next, a significant inhibition of apoptosis was observed in MEHHP exposed MYOF organoid when compared to either the untreated control or MEHHP exposed MYON organoids (*p* < 0.001) (Figure 2A,B). This was accompanied by a significant reduction in Caspase3 protein and gene levels in the MEHHP‐exposed MYOF organoids as compared to MEHHP exposed MYON organoids (each was normalized to its own VEH exposed group), as demonstrated by IHC (*p* < 0.0001) (Figure 2C,D) and RT‐PCR (*p* < 0.01) (Figure 2E).

**FIGURE 3:**
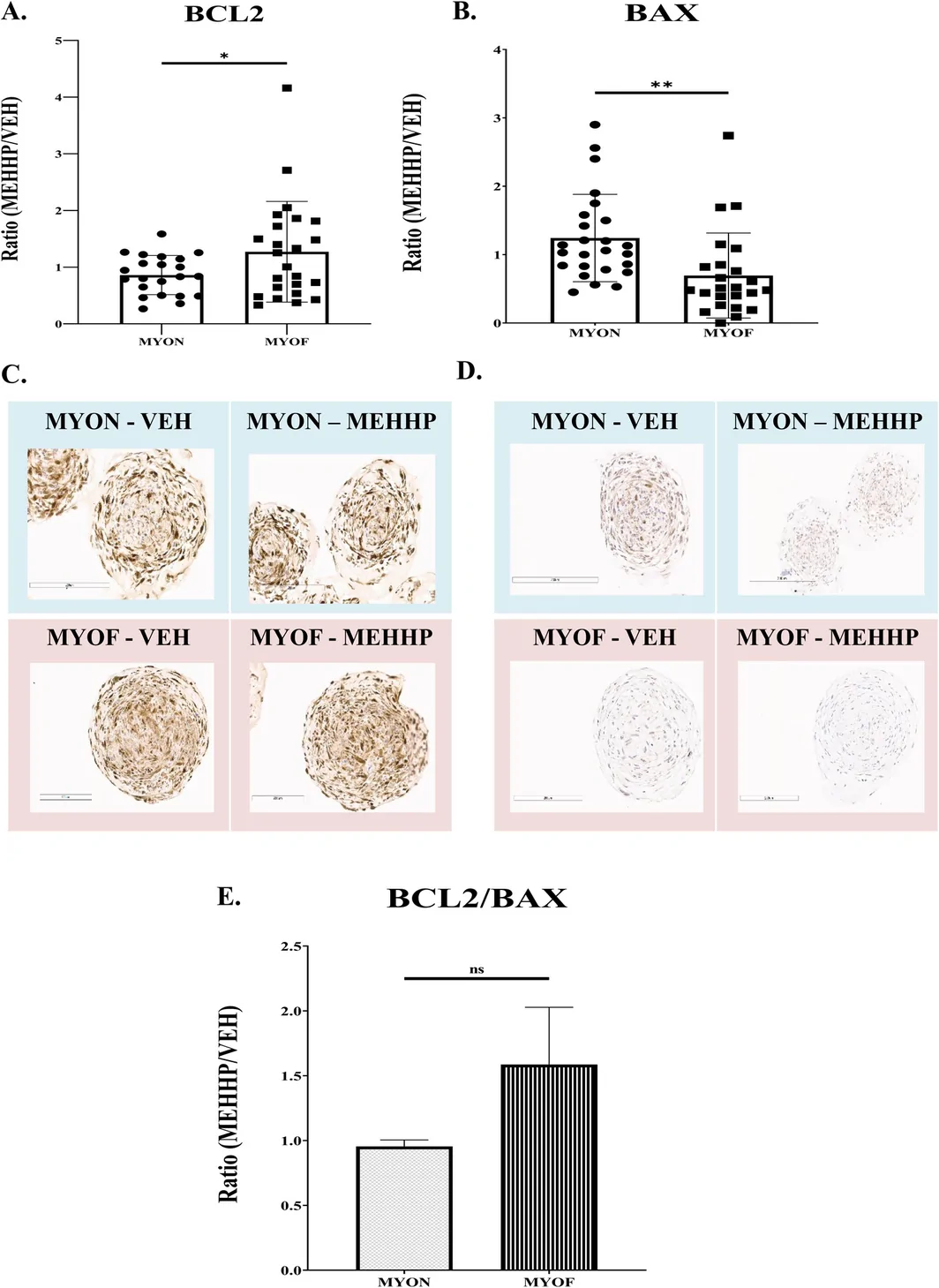
3D organoid MEHHP exposed leads to impaired apoptosis. (A, C) Compared to MEHHP‐exposed MYON organoids, MEHHP‐exposed MYOF organoids exhibit an elevated expression of BCL2, indicating a shift towards pro‐survival signaling and a potential increase in cell survival following an apoptotic stimulus via IHC analysis. (B, D) MEHHP‐exposed MYOF organoids lead to a notable decrease in BAX expression, suggesting a reduction in apoptotic activity and indicating a potential suppression of programmed cell death via IHC analysis. (E) MEHHP‐exposed MYOF organoids induce a significant increase in the expression of BCL2/BAX, suggesting a shift towards a more pro‐survival phenotype and a potential enhancement of cellular resistance to apoptotic stimuli via RT‐PCR analysis. Error bars represent the mean + s.d. *p < 0.05; **p < 0.01; ***p < 0.001. n.s., no significant difference. *p* values were analyzed by unpaired two‐tailed Student's *t*‐test and two‐way ANOVA.

Similarly, MEHHP exposure led to a significant increase in BCL2 protein levels (Figure 3A,C), whereas decreased BAX protein levels in MYOF versus MYON (Figure 3B,D) based on IHC analysis. Consistently, RT‐PCR analysis showed a similar pattern with an increased *BCL2* and a decreased *BAX* gene expression (*p* < 0.01) and subsequent increased *BCL2/BAX* ratio (Figure 3E). These findings highlight the potential impact of MEHHP exposure on apoptosis‐related proteins, indicating a potential role in modulating cellular survival pathways in MYOF organoids in comparison with MYON towards UF phenotype.

### 3.2 MEHHP Exposure Results in More ECM Accumulation in At‐Risk MYOF Organoids

**FIGURE 4:**
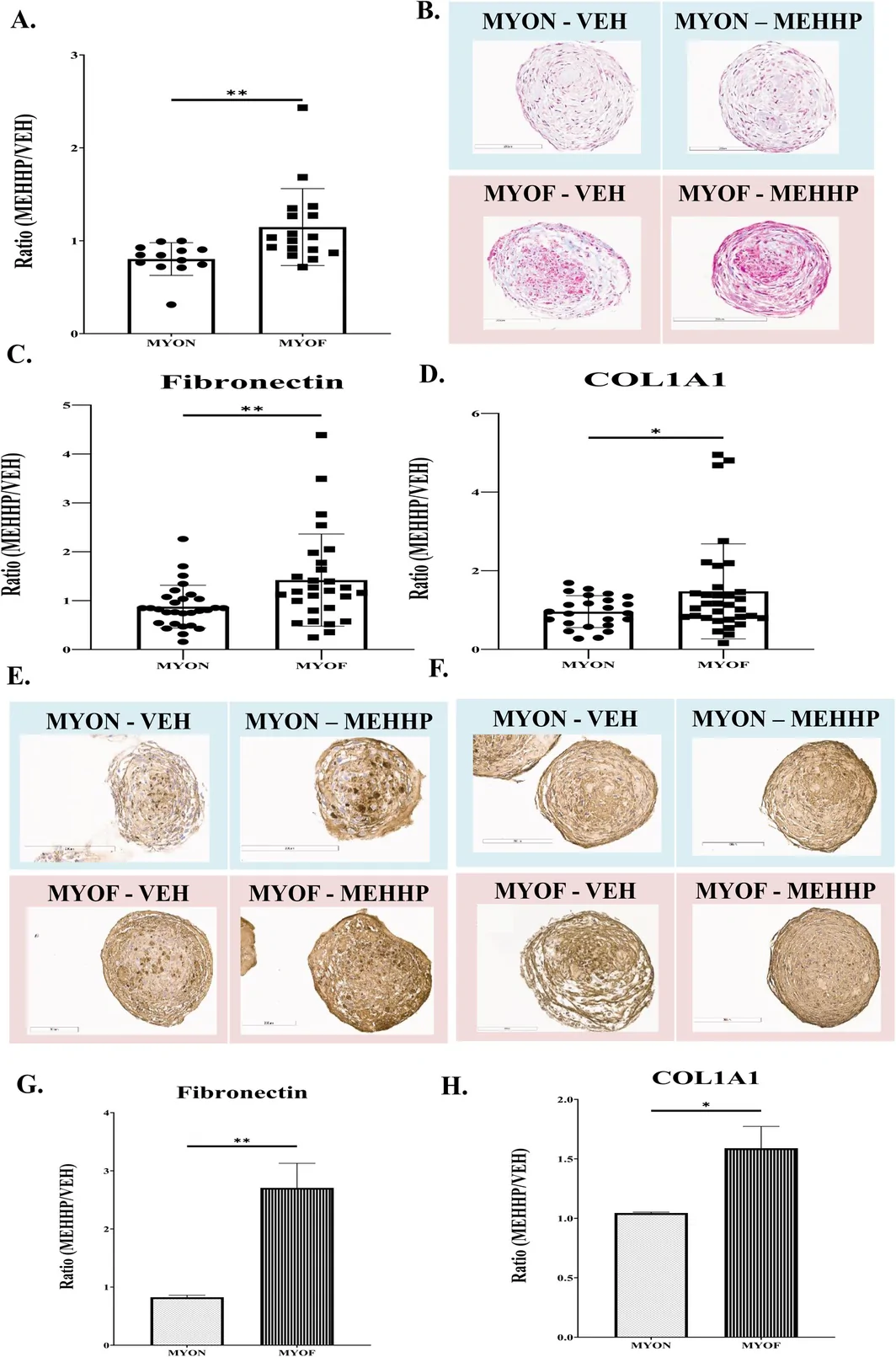
MEHHP exposure results in an overexpression of ECM transition. (A, B) MEHHP exposure to MYOF organoids leads to an increase in the production or deposition of ECM protein as evidenced by trichrome staining compared to MYON organoids (C, E) MEHHP‐exposed MYOF organoids showed an upregulation of Fibronectin via IHC analysis. (D, F) MEHHP‐exposed MYOF organoids showed an upregulation of COL1A1 expression compared to MEHHP‐exposed MYON organoids, via IHC analysis. (G, H) MEHHP Exposure to MYOF organoids results in a significant elevation in FN1 and COL1A1 gene expression, via the RT‐PCR analysis compared to MyoN organoids. Error bars represent the mean + s.d. *p < 0.05; **p < 0.01; ***p < 0.001. n.s, no significant difference. *p* values were analyzed by unpaired two‐tailed Student's *t*‐test and two‐way ANOVA.

MEHHP exposure to MYOF organoids led to a significant increase in Trichome staining compared to MEHHP exposed MYON organoids (each was normalized to its own VEH exposed group) (*p* < 0.001) (Figure 4A,B). Notably, MEHHP exposure resulted in elevated expression levels of Fibronectin (*p* < 0.001) and COL1A1 (p < 0.01) proteins, as demonstrated through IHC staining (Figure 4C,D,E,F), respectively. Consistent with these findings, RT‐PCR analysis revealed upregulated gene expression levels of *FN* and *COL1A1* in response to MEHHP exposure (Figure 4G,H). The increased Trichome staining indicates potential alterations in ECM components, suggesting an impact on tissue structure and composition in the organoids exposed to MEHHP. The augmented expression of FN and COL1A1 proteins, along with the corresponding upregulated gene expression, suggests a potential role of MEHHP in modulating the synthesis of ECM proteins, which are crucial for cellular adhesion, migration, and tissue homeostasis [71, 72]. These findings provide insights into the molecular changes induced by MEHHP in MYOF and suggest a possible involvement of this compound in tissue remodeling processes.

### 3.3 MEHHP Exposure Triggers Cytokines Secretion Towards Inflamed Myometrium in At‐Risk MYOF Organoids

**FIGURE 5:**
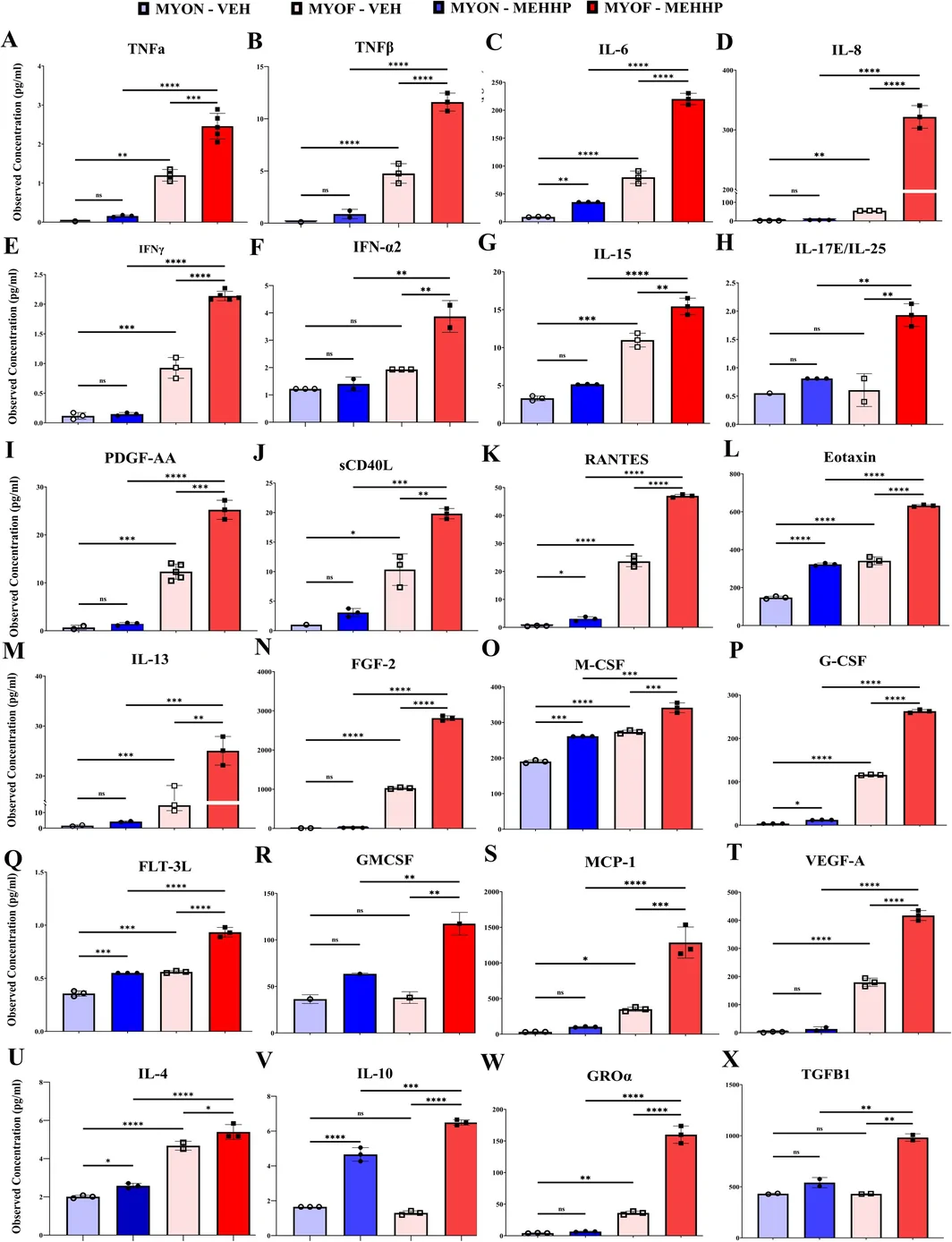
MEHHP exposure induces cytokines production towards an inflamed myometrium. The production of various cytokines and growth factors is increased in MYOF organoids condition media following MEHHP exposure as compared to MYON organoids, using Multiplex Cytokine Array. Specifically, the levels of TNFα (A), TNFβ (B), IL‐6 (C), IL‐8 (D), IFNδ (E), IFN‐α2 (F), IL‐15 (G), IL‐17E/IL‐25 (H), PDGF‐AA (I), sCD40L (J), RANTES (K), Eotaxin (L), IL‐13 (M), FGF‐2 (N), M‐CSF (O), G‐CSF (P), FLT‐3L (Q), GMCSF (R), MCP‐1 (S), VEGF‐A (T), IL‐4 (U), IL‐10 (V), GROα (W), TGFB1 (X) are markedly increased, indicating an exaggerated response in the MEHHP‐exposed MYOF organoids. Error bars represent the mean + s.d. *p < 0.05; **p < 0.01; ***p < 0.001. n.s. no significant difference. *p* values were analyzed by unpaired two‐tailed Student's *t*‐test and two‐way ANOVA.

The exposure to MEHHP has been found to exert a profound influence on the cytokines and growth factors secretion from MYOF organoids compared to those derived from MYON using comprehensive multiplex Cytokine Array analysis with elevated levels of pro‐inflammatory cytokines, namely TNFα, TNFβ, IL‐6, and IL‐8 (Figure 5A–D). These findings provide compelling evidence of an activated inflammatory state within the MYOF organoids upon exposure to phthalate, suggesting a potential link between these cytokines and the development and progression of UF [73, 74]. Additionally, MEHHP exposure showed a significant impact on other immune‐related cytokines, such as IFN‐α2 and IFNγ (Figure 5E,F). These cytokines are known to play pivotal roles in antiviral responses and neutrophil recruitment, indicating potential implications for the immune response within the myometrial organoids. Furthermore, the elevated levels of IL‐15 and IL‐17E/IL‐25 (Figure 5G,H) suggest significant effects on immune cell development and regulation, which could profoundly impact natural killer (NK) cells, CD8+ T cells, and the differentiation of Th17 and Th2 cells.

Remarkably, MEHHP exposed MYOF organoids exhibit higher levels of certain growth factors compared to MEHHP exposed MYON organoids. These growth factors include PDGF‐AA, sCD40L, RANTES, and Eotaxin (Figure 5I–L). Such differential expression of these growth factors may significantly influence cell signaling and chemotaxis within the organoids. PDGF‐AA is known to promote cell migration, proliferation, and angiogenesis [75], whereas sCD40L and RANTES play vital roles in immune cell activation and recruitment, contributing to altered cellular responses and tissue remodeling in the presence of phthalate metabolites [76, 77]. Furthermore, Eotaxin's involvement suggests a key role in recruiting eosinophils, a type of immune cell crucial in allergic responses and inflammation. In the context of UF growth, chemokines and their corresponding receptors play a significant role. Notably, MIP‐1α and MIP‐1β, along with RANTES, Eotaxin, Eotaxin‐2, IL‐8, CCR1, CCR3, and CCR5, as well as CXCR1 and CXCR2, and finally MCP‐1, which are involved in stimulating UF growth through their interaction with estrogens and progesterone [78, 79].

Among the cytokines affected in response to MEHHP exposure is IL‐13 (Figure 5M), with its established role in modulating immune responses and regulating inflammatory processes [80], where its secretion was increased in MYOF versus MYON organoids. This finding indicates a potential impact of MEHHP exposure on immune regulation and inflammation in these organoids.

Likewise, FGF2, a factor recognized for its role in cell proliferation and tissue repair (Figure 5N) [81] was highly secreted from MYOF organoids compared to MYON after MEHHP exposure. This suggests a direct influence of MEHHP exposure on FGF‐2 in these at‐risk organoids, which may have implications for cellular proliferation and tissue repair processes towards UF phenotype.

MEHHP exposure also had a considerable effect on the expression levels of M‐CSF (Figure 5O) and G‐CSF (Figure 5P). M‐CSF and G‐CSF are known to stimulate the production and differentiation of immune cells, specifically macrophages and granulocytes, respectively [82]. The significant changes in their expression levels suggest that MEHHP exposure may have a substantial impact on the regulation of these immune cell types within the organoids.

Similarly, FLT‐3L, a growth factor involved in the production of dendritic cells and hematopoietic stem cells [83], exhibited significant changes in its expression levels upon MEHHP exposure (Figure 5Q). This indicates that FLT‐3L may maintain its regulatory role in immune cell development despite the presence of phthalate metabolites.

Additionally, GM‐CSF exhibited a significant increase in its expression levels (Figure 5R), especially in MYOF organoids after MEHHP exposure. GM‐CSF plays a vital role in promoting the growth and differentiation of granulocytes and macrophages. The observed alterations in GM‐CSF levels suggest potential regulation of immune cell populations, influencing immune responses and tissue remodeling processes in response to MEHHP. Moreover, exposure to MEHHP resulted in noteworthy changes in MCP‐1 expression levels (Figure 5S). MCP‐1 is crucial for recruiting monocytes to sites of inflammation and tissue injury. This implies that MEHHP exposure in MYOF organoids may significantly impact the recruitment of monocytes, thereby influencing immune responses and tissue repair mechanisms as compared to MYON organoids. Improper regulation of macrophage proliferation, accumulation, and infiltration could lead to uncontrolled tissue repair and subsequent pathological fibrosis. Additionally, MCP‐1, GM‐CSF, TGF‐β, activin A, and TNF‐α are implicated in macrophage actions during uncontrolled tissue repair, contributing to the pathological fibrosis as a hallmark of UF pathogenesis [84].

Notably, MEHHP exposure to MYOF organoids induced exaggerated upregulation of VEGF‐A secretion, compared to MYON organoids (Figure 5T). VEGF is a master regulator of neovascularization and has been found to be elevated in patients with UF [85]. In fact, the increased HIF‐1α expression in fibroid cells may nevertheless lead to upregulation of several downstream genes involved in angiogenesis, including VEGF‐A [86]. Moreover, the expression levels of IL‐4 and IL‐10 were significantly affected by MEHHP exposure (Figure 5U,V). Both cytokines are involved in anti‐inflammatory responses and immune regulation [87], and their alterations suggest significant modulation of anti‐inflammatory pathways within the organoids under the influence of MEHHP.

Additionally, the levels of CXCL1/GROα and TGFβ1 showed significant changes upon MEHHP exposure (Figure 5W,X). GROα plays a role in inflammation and cell migration. High expression level of GROα is linked to advanced stage and worse survival in uterine cervical cancer and facilitates tumor cell malignant processes [88]. TGF‐β is a multifunctional cytokine involved in regulating cell growth and differentiation and one of the key factors involved in the pathophysiology of UF. It involves cellular migration within the tumor, stimulates tumor growth, and enhances tumor metabolism [89]. The increased expression of these cytokines in response to MEHHP exposure suggests that the phthalate may have substantial impact on their functions in the organoids, influencing cellular responses and tissue remodeling.

To sum up, the exposure to MEHHP induces a significant increase in several cytokines and growth factors in MYOF organoids compared to MYON organoids, whereas others remain relatively unaffected. These findings provide valuable insights into the complex interplay of cytokine responses upon phthalate exposure and their potential implications for UF development and pathogenesis. Further research is warranted to elucidate the precise underlying mechanisms and explore the specific roles of these cytokines and growth factors in the context of fibroid biology. Understanding the impact of MEHHP exposure on cytokine expression profiles is crucial for developing targeted therapeutic interventions aimed at mitigating fibroid‐related risks and improving clinical outcomes for affected individuals.

### 3.4 RNA‐Seq Analysis Uncovers an Elevated Presence of OXPHOS in MYOF Organoids in Response to MEHHP Exposure

Since RNA‐seq is a transformative tool that has significantly advanced understanding of gene function, molecular mechanisms, and disease pathogenesis [90, 91]. In the context of our research, profiling the transcriptome of our organoids in response to MEHHP exposure can provide a comprehensive view of gene expression patterns which can be involved in UF pathogenesis [92]. RNA‐seq enables shedding light on the intricate regulatory networks and molecular mechanisms underlying organoids which are exposed to MEHHP [93]. RNA‐Seq analysis was performed on four groups, including MYOF‐VEH, MYOF‐MEHHP, MYON‐VEH, and MYON‐MEHHP. Biological replicates were used to ensure experiment validity. Evaluating gene expression correlations between samples helped verify reliability and proper sample selection, aiding in differential gene expression analysis. The correlation coefficient, a measure of sample similarity, was critical for assessing data quality, and an ideal experiment condition required a coefficient square > 0.92 and R2 > 0.8. The correlation coefficient matrix was represented in Figure S1B. Differentially expressed genes (DEGs) from comparison groups were combined to create the differential gene set. Hierarchical clustering with row homogenization (*Z*‐score) of FPKM values was used to group genes with similar expression patterns.

In the first comparison, we compared the MYOF‐MEHHP group versus the MYON‐MEHHP group. The co‐expression Venn diagram visually depicted the genes that were exclusively expressed within each group and those that were co‐expressed in multiple groups. Notably, the MYOF‐MEHHP group and the MYON‐MEHHP group shared 9122 genes and 10 061 genes, respectively (Figure S2A,B). Among these genes, 3064 were upregulated, whereas 2009 were downregulated (Figure S2C). It's worth noting that the mitochondrial inner membrane category accounted for the largest gene count and gene ratio. Regarding the GO enrichment analysis for Biological Processes (BP), OXPHOS exhibited statistical significance. Furthermore, in Cellular Components (CC), the mitochondrial protein complex displayed significance, whereas NADH dehydrogenase activity was identified as an important Molecular Function (MF) (Figure S2*D). In the KEGG pathway analysis, we observed a high expression of OXPHOS (Figure S2E). Additionally, pathways related to respiratory electron transport, ATP synthesis by chemiosmotic coupling, and heat production by uncoupling proteins were notably significant in Reactome (Figure 2F). Moreover, the DisGeNET database indicated a higher occurrence of mitochondrial respiratory chain defects in this comparison.

We then proceeded with the comparison between the MYOF‐MEHHP group and the MYOF‐VEH group. The Venn diagram showed an overlap of 9122 genes between these two groups, and the MYOF‐VEH group had an additional 8906 unique genes (Figure S3*A,B). Among these genes, 915 showed upregulation, and 652 displayed downregulation (Figure S3C). We found that intermediate filaments were particularly significant in CC based on GO enrichment analysis (Figure S3D). Moreover, the KEGG pathway analysis revealed high expression levels in both OXPHOS and thermogenesis (Figure S3E). Reactome pathways also highlighted the significance of respiratory electron transport, ATP synthesis by chemiosmotic coupling, and heat production by uncoupling proteins (Figure S3F).

The third comparison involved the MYON‐MEHHP group versus the MYON‐VEH group. Here, the MYON‐MEHHP group exhibited 10 061 genes in common with the MYON‐VEH group, whereas the latter group had 8997 unique genes (Figure S4A,B). Among these genes, 478 showed upregulation, and 826 displayed downregulation (Figure S4C). GO enrichment analysis indicated that BP related to extracellular matrix organization was statistically significant. Moreover, CC was enriched with extracellular matrix components, and receptor regulator activity played an important role as a MF (Figure S4D). In the KEGG pathway analysis, Focal adhesion was highly expressed (Figure S4E), and Reactome pathways highlighted the significance of ECM (Figure S4F). Interestingly, the DisGeNET database revealed an upregulation of genes associated with Pulmonary Fibrosis in this comparison.

Finally, we explored the comparison between the MYOF‐VEH group and the MYON‐VEH group. These groups shared 8906 and 8997 genes, respectively (Figure S5A,B), with 856 genes upregulated and 794 genes downregulated (Figure S5C). According to GO enrichment analysis, BP related to reproductive system development exhibited statistical significance. Additionally, the CC was significantly enriched with the ECM, and glycosaminoglycan binding played a vital role as a MF (Figure S5D). KEGG analysis did not show any significant pathway (Figure S5E). Reactome pathways suggested higher expression levels in IL‐4 and IL‐13 signaling pathways (Figure S5F). Moreover, the DisGeNET database indicated an association with inflammation.

### 3.5 Unveiling Distinct Oxidative Stress (OS) Gene and Protein Expression Patterns in MYOF Organoids in Response to MEHHP Exposure

In this study, we conducted a thorough analysis in GO and KEGG for the common genes shared between the MYOF‐MEHHP and MYON‐MEHHP experimental groups, as depicted in Figure S6A. Notably, these genes were not differentially expressed in the comparison between the MYOF‐MEHHP and MYOF‐VEH groups, as shown in Figure S6B. Subsequently, we identified 19 genes that were uniquely expressed in the MYOF‐MEHHP group, distinguishing them from both the MYON‐MEHHP and MYOF‐VEH groups (Figure S6C and Table S1). Building upon this observation, we proceeded to investigate the GSEA for the comparison of MYOF‐MEHHP versus MYON‐MEHHP. The results from the GSEA analysis highlighted the significance of OXPHOS in this particular comparison, as depicted in Figure S6D. Based on these compelling findings, we carefully selected six genes for further investigation, as illustrated in Figure S6E.

**FIGURE 6:**
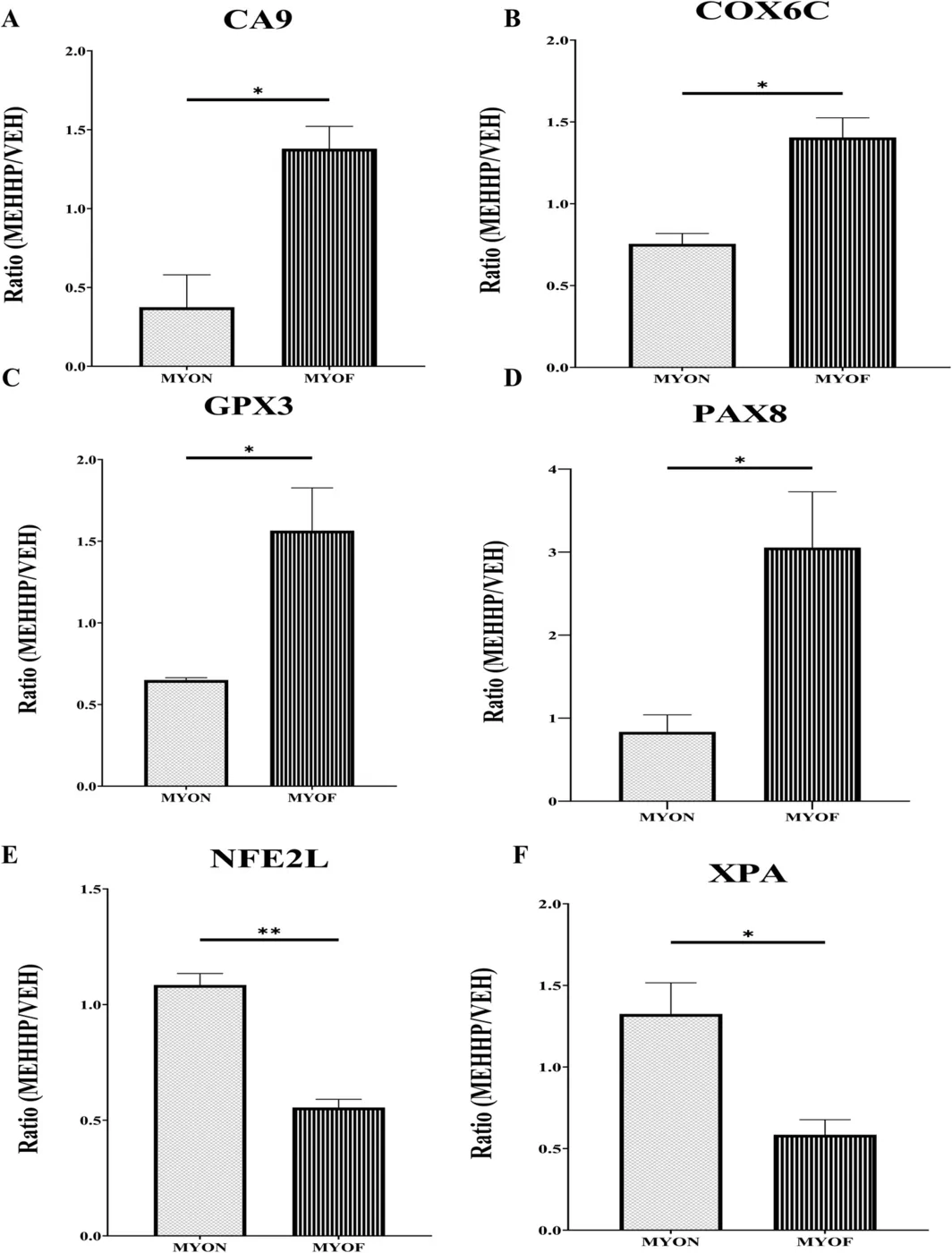
MEHHP exposure induced oxidative stress (OS) in MYOF organoids using RT‐PCR analysis. MEHHP‐exposed MYOF organoids, compared to MEHHP‐exposed MYON organoids, exhibited higher gene expression levels of (A) CA9, a gene associated with oxidative stress (OS) regulation, (B) PAX8, known for its role in transcriptional regulation and cell differentiation, (C) GPX3, known for its role in antioxidant defense, (D) COX6C, a gene associated with cellular respiration and mitochondrial function. Although MEHHP exposure resulted in decreased gene expression of (E). The transcription factor NFE2L, which participates in cellular responses to oxidative stress (OS), and (F). XPA, known for its role in DNA repair, in MEHHP‐exposed MYOF organoids, compared to MEHHP‐exposed MYON organoids. Error bars represent the mean + s.d. *p < 0.05; **p < 0.01; ***p < 0.001. No significant difference. *p* values were analyzed by unpaired two‐tailed Student's *t*‐test and two‐way ANOVA.

Among these genes, *CA9* is associated with OS regulation, exhibited higher gene expression levels in MEHHP‐exposed MYOF organoids compared to MEHHP‐exposed MYON organoids (Figure 6A). This observation suggests that at‐risk myometrial cells might experience elevated OS levels, potentially linked to their involvement in tissue repair and remodeling processes. The *PAX8*, known for its role in transcriptional regulation and cell differentiation, displayed high expression levels in MEHHP‐exposed MYOF organoids in contrast to MEHHP‐exposed MYON organoids (Figure 6B). Additionally, *GPX3*, known for its role in antioxidant defense, exhibited higher expression levels in MEHHP‐exposed MYOF organoids compared to MEHHP‐exposed MYON organoids (Figure 6C). This upregulation of GPX3 in myometrium suggests a protective mechanism against the increased OS they encounter during tissue repair processes. Furthermore, the presence of COX6C, a protein associated with cellular respiration and mitochondrial function, was more abundant in MEHHP‐exposed MYOF organoids than in MEHHP‐exposed MYON organoids (Figure 6D). This finding indicates that they may have a higher demand for energy production through OXPHOS, possibly due to their role in wound healing and fibrosis.

Contrastingly, the transcription factor NFE2L, which participates in cellular responses to OS, was downregulated in MEHHP‐exposed MYOF organoids compared to MEHHP‐exposed MYON organoids (Figure 6E). This observation raises questions about the differences in OS response mechanisms between these two organoid types. Lastly, the protein XPA, known for its role in DNA repair, exhibited lower expression levels in MEHHP‐exposed MYOF organoids than in MEHHP‐exposed MYON organoids (Figure 6F). This discrepancy suggests variations in DNA repair capacity between the two organoid types, possibly reflecting their distinct cellular functions. Subsequently, we conducted a comprehensive analysis of gene expression and protein signatures in MEHHP‐exposed MYOF and MEHHP‐exposed MYON organoids, with a specific focus on proteins associated with cellular responses to OS and DNA repair.

**FIGURE 7:**
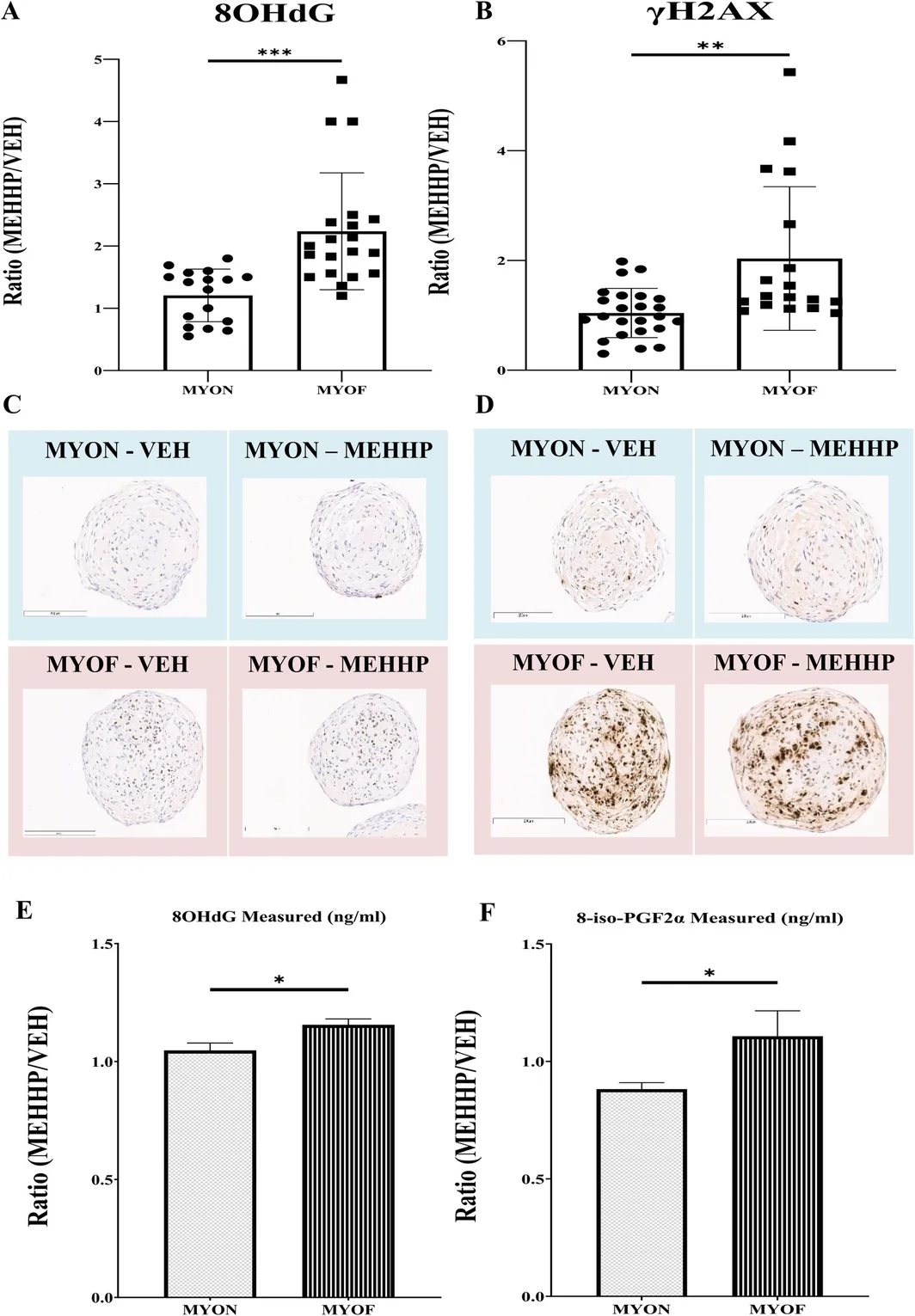
MEHHP exposure induced oxidative stress (OS) & DNA Damage accumulation in MYOF organoids compared to MYON organoids. (A, C) IHC representative images and analysis of 8‐OHdG measurement, a marker of oxidative stress (OS), showing a statistically higher ratio in MEHHP exposed MYOF organoids compared to MEHHP‐exposed MYON organoids. (B, D) IHC representative images and analysis of DNA damage marker γH2AX expression, indicating an upregulation in MYOF organoids in response to MEHHP exposure compared to MYON organoids. (E, F) The results of ELISA analysis, showing significantly higher secretion of 8OHdG and 8‐iso‐PGF2α expression from MEHHP‐exposed MYOF organoids compared to MEHHP‐exposed MYON organoids. Error bars represent the mean + s.d. *p < 0.05; **p < 0.01; ***p < 0.001. n.s., no significant difference. *p* values were analyzed by unpaired two‐tailed Student's *t*‐test and two‐way ANOVA.

Our findings revealed significant disparities in protein expression between MEHHP‐exposed MYOF and MEHHP‐exposed MYON organoids. Among the investigated proteins, Figure 7A,C demonstrated the measurement of 8‐OHdG, a well‐established marker of OS, using IHC analysis. Interestingly, MEHHP exposure to MYOF organoids resulted in significantly higher expression of 8‐OHdG compared to MEHHP‐exposed MYON organoids, each normalized to its VEH exposure, indicating an elevated level of OS.

Furthermore, Figure 7B,D displayed the results of IHC analysis, revealing an upregulation of γH2AX expression in MEHHP‐exposed MYOF organoids compared to MEHHP‐exposed MYON organoids. This observation points to the accumulation of DNA damage, which can be attributed to MEHHP exposure, suggesting a potential role of MEHHP in influencing DNA repair mechanisms specifically in MYOF organoids. Additionally, Figure 7E depicted the outcomes of ELISA analysis, which showed a significant increase in the expression of 8OHdG in MEHHP‐exposed MYOF organoids. This finding corroborates the occurrence of oxidative damage to DNA induced by MEHHP, providing further evidence of its impact on DNA integrity and repair processes. Moreover, Figure 7F presented the results of ELISA analysis, demonstrating a pronounced upregulation of 8‐iso‐PGF2α expression in MEHHP‐exposed MYOF organoids. This elevation in 8‐iso‐PGF2α levels serves as an indicator of heightened OS in response to MEHHP. 8‐iso‐prostaglandin F2α is a type of prostaglandin, which is a lipid compound derived from fatty acids. Prostaglandins are involved in various physiological processes and can have both pro‐inflammatory and anti‐inflammatory effects [53, 94].

The observed differences in protein expression and the increased OS response in MEHHP‐exposed MYOF organoids provide valuable insights into the potential role of MEHHP in the development and progression of UF. OS has been implicated in various pathological processes, including fibrosis and tissue remodeling [95], suggesting that the elevated OS levels in MEHHP‐exposed MYOF organoids may contribute to the fibrotic characteristics associated with UF.

Moreover, the accumulation of DNA damage in MEHHP‐exposed MYOF organoids raises intriguing questions about the potential impact of MEHHP on DNA repair mechanisms, which could have significant implications for the genomic stability of UF cells. These findings provide a deeper understanding of the molecular mechanisms underlying UF pathogenesis and offer potential avenues for further research and therapeutic interventions.

The correlation between 8‐OHdG and γH2AX can provide insights into the relationship between oxidative stress (OS)‐induced DNA damage and the activation of DNA repair mechanisms in UF to be further explored. It helps to understand the interplay between OS and DNA damage response pathways and their implications for cellular homeostasis, aging, and disease development. Increased levels of both 8‐OHdG and γH2AX have been observed in conditions associated with OS and DNA damage, such as exposure to environmental toxins, radiation, or certain diseases [53, 96].

### 3.6 MEHHP Exposure Induced Mitochondrial Dysfunction in MYOF Organoids

The intricate relationship between mitochondria and apoptosis, a programmed cell death process, is of significant interest in understanding the pathogenesis of UF. Dysfunctional mitochondria, particularly with impaired OXPHOS, can disrupt the delicate balance between pro‐apoptotic and anti‐apoptotic factors, leading to alterations in the apoptotic signaling pathway. Mounting evidence suggests that alterations in mitochondrial DNA copy number (mtDNAcn) may play a crucial role in UF development [97].

**FIGURE 8:**
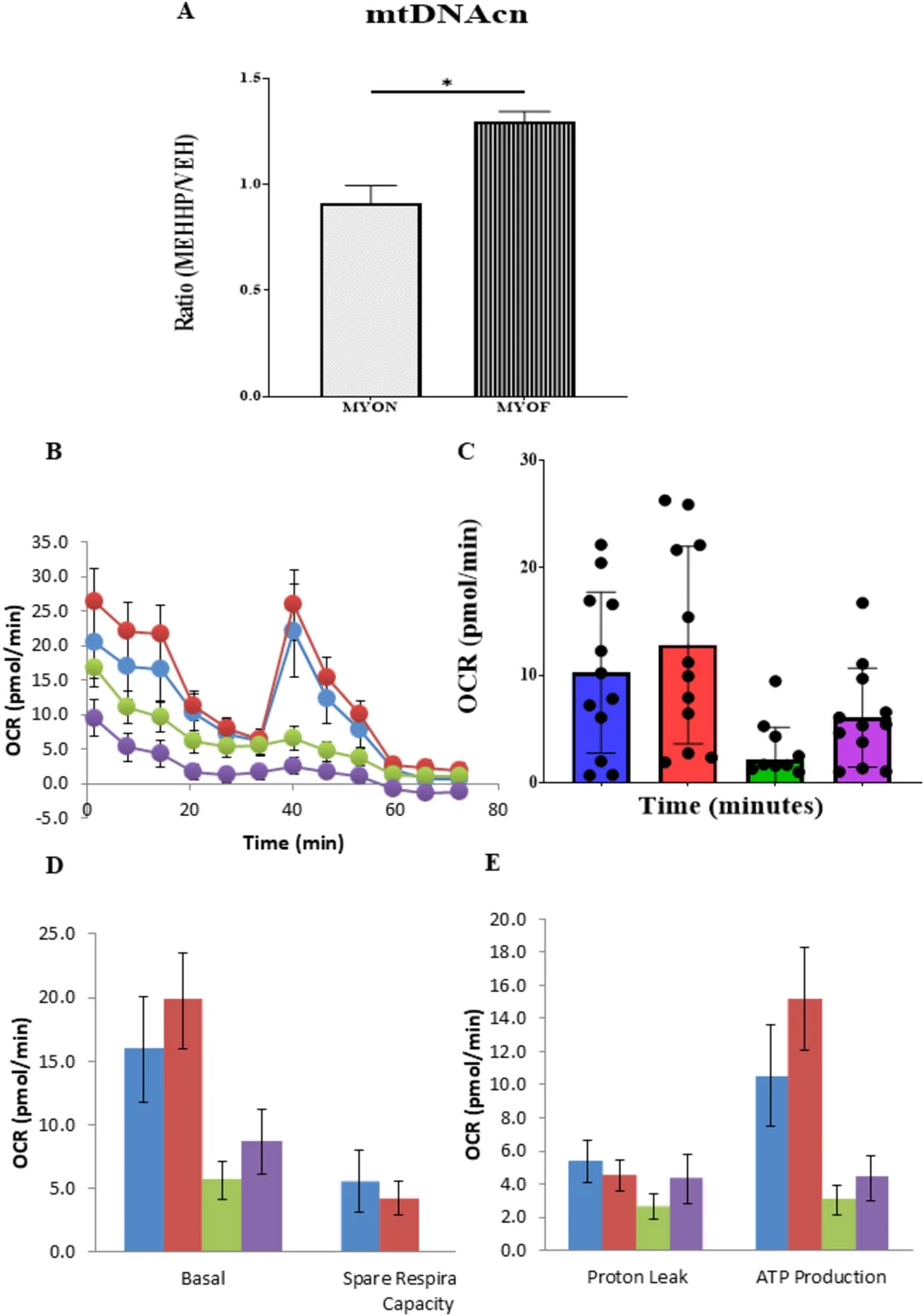
Mitochondrial dysfunction in myometrial organoids Induced by MEHHP exposure. (A) MEHHP‐exposed MYOF organoids exhibit increased mtDNA replication, potentially contributing to the observed mitochondrial dysfunction compared to MYON organoids. (B, C) Representative traces of Oxygen Consumption Rate (OCR) (pmol) (min/μg protein) which are increased showcased distinct metabolic profiles between MEHHP‐exposed MYOF and MYON organoids by XFe96 extracellular flux analysis (*n* = 4) as compared to MYON organoids. (blue: MYOF‐VEH, red: MYOF‐MEHHP, green: MYON‐VEH, and purple: MYON‐MEHHP). (D, E) Mitochondrial oxygen consumption were generated, displaying the averages ± standard deviations for each measurement time point. The initial three measurement points represented the basal mitochondrial respiration. Following this, the next three points demonstrated proton leak‐stimulated oxygen consumption after blocking adenine nucleotide translocating with the addition of oligomycin. The subsequent three points represented the maximal mitochondrial oxygen consumption capacity, achieved when the mitochondrial inner membrane was uncoupled by FCCP (carbonyl cyanide‐4 (trifluoromethoxy) phenylhydrazone). The last three points represented non‐mitochondrial oxygen consumption, which occurred when the mitochondrial respiratory chain was inhibited by rotenone and antimycin Analysis of functional integrity of mitochondria, Data extracted from the analyses in C.

Here, we focused on investigating the mtDNAcn alterations in MYOF organoids as compared to MYON organoids after MEHHP exposure, to shed light on the potential impact of phthalate on mitochondrial dysfunction and thus fibroid pathogenesis. We observed an upregulation of the mtDNA‐to‐nuclear DNA (nDNA) ratio in MEHHP‐exposed MYOF organoids, as depicted in Figure 8A. This finding suggests that MEHHP exposure to at‐risk MYOF organoids induced aberrant mitochondrial biogenesis, as shown by increased mtDNA replication, potentially contributing to the observed mitochondrial dysfunction compared to normal MYON organoids.

Proper mitochondrial function is essential for the activation of caspases and the release of apoptotic factors during apoptosis. Dysregulated mitochondrial activity may influence the apoptotic signaling cascade, potentially impacting cell survival and proliferation in UF. The observed alterations in mitochondrial function in MEHHP‐exposed MYOF organoids raise intriguing questions about their potential involvement in the apoptotic resistance commonly observed in fibroid tissues.

To comprehensively evaluate mitochondrial function, we conducted the Seahorse XFp Cell Mito Stress Test Kit, which measures oxygen consumption rate (OCR). This analysis provided valuable insights into the bioenergetic profile and metabolic state of the organoids, which is illustrated in Figure 8B,C, and enabled us to assess the mitochondrial respiration capacity and glycolytic activity in the four experimental groups. Where both VEH‐exposed and MEHHP‐exposed MYOF organoids showed higher OCR compared to VEH‐exposed and MEHHP‐exposed MYON organoids, correspondingly with MEHHP‐exposed MYOF organoids showing highest OCR (Figure 8B,C). The OCR results, depicted in Figure 8D,E, showcased distinct metabolic profiles between MEHHP‐exposed MYOF and MEHHP‐exposed MYON organoids. The observed differences in mitochondrial respiration and glycolytic activity may underpin altered energy metabolism in fibroid cells, potentially influencing their survival and proliferation rates. Such increasingly metabolic activity is recognized as a hallmark of tumorigenesis, and understanding its implications in UF is of paramount importance. The higher level of OCR indicates more active respiration that is observed in MEHHP‐exposed MYOF organoids.

By unraveling the complex interplay between mitochondrial dysfunction and apoptotic signaling, our study contributes to the growing body of literature exploring the molecular mechanisms driving UF development and progression. The observed alterations in mtDNAcn, mitochondrial activity, and metabolic profile offer valuable insights into the potential therapeutic targets and strategies for managing UF‐related symptoms.

## 4 Discussion

The burden of UF in the United States is substantial, costing an estimated annual sum of up to $34 billion [98], with a disproportionate impact on black women [99]. Current treatment strategies rely mainly on surgical or radiological interventions [100] that compromise reproductive potential, with limited medical options and no effective prevention strategies [101].

A major barrier to prevention is the incomplete understanding of UF etiology and modifiable risk factors [102]. Although early‐life exposure to EDCs, like DES, has been recognized as a significant risk factor for fibroids, our knowledge about other environmental EDCs, particularly phthalates, including MEHHP metabolites, remains underexplored despite their widespread exposure among women [23, 103].

Our previous work demonstrated that early‐life EDC exposure epigenetically reprograms myometrial mesenchymal stem cells (MMSCs), rendering them more susceptible to fibroid formation through TGFβ pathway hyperactivation, impaired Nucleotide Excision Repair (NER), and increased genetic instability [29, 30, 43]. MMSCs‐derived organoids offer several advantages, such as rapid establishment, robustness, potential for genetic manipulation using modern genetic engineering tools, and personalization based on individual samples [32, 104]. These epigenetic alterations mediated by factors such as Mixed Lineage Leukemia 1 (MLL1) and Histone Deacetylase (HDAC) lead to a persistent “hyper‐inflammatory” and “hyper‐estrogenic” phenotype in MMSC, increasing their vulnerability to environmental “second hits” [14, 105, 106].

In our current study, we utilized our 3D MMSC‐derived organoid model to investigate the effects of MEHHP exposure as a potential risk factor of UF development via induction of a second hit in at‐risk MYOF organoids, as compared to MYON organoids, as evidenced by upregulation of several UF related phenotype markers such as ECM, proliferation, impaired apoptosis, inflammation, DNA damage accumulation, and more OS. Previous findings had already highlighted the significance of phthalate‐associated miRNAs and their mRNA gene targets, which were linked to various fibroid‐related processes, including angiogenesis, apoptosis, and proliferation of connective tissues [107]. Another research team demonstrated that MEHHP promotes the survival of UF cells by affecting tryptophan uptake, kynurenine production, and activating the aryl hydrocarbon receptor pathway [23].

MEHHP‐induced ECM remodeling was evidenced by increased trichrome staining and elevated FN and COL1A1 expression, reflecting the structurally abnormal ECM previously implicated in fibroid growth. Understanding the critical role of ECM stiffness in fibroid growth may lead to new strategies for treating this common disease [51]. Previous research identified unique ECM proteins present only in fibroids, regardless of *MED12* mutation [108], that confirm the important role ECM plays in UF pathogenesis.

MEHHP also triggered a hyper‐inflammatory response characterized by elevated TNFα, TNFβ, and IL‐6 levels, along with other immune mediators such as IFN‐α2, IL‐8, IL‐15, IL‐17E/IL‐25, and PDGF‐AA [109]. These cytokines and growth factors regulate immune cell recruitment, proliferation, and tissue remodeling, indicating that MEHHP exposure actively shapes an inflammatory and fibrotic microenvironment [110]. Additional cytokines (e.g., IL‐13, GROα, MCP‐1, GM‐CSF, TGFβ1) further underscore this coordinated immune–ECM response. The interplay between inflammation and OS is a well‐established driver of UF progression [111].

The exposure to MEHHP had a profound and significant impact on the cytokine secretory profile of MYOF organoids. Notably, there were distinct differences observed between MYOF and MYON organoids in response to this exposure. The affected cytokines are key players in immune regulation, tissue repair, and growth control, indicating the involvement of multiple critical cellular processes in the organoids' response to the phthalate exposure. The observed alterations in cytokine levels strongly suggest a deliberate and targeted modulation of immune responses within the organoids as a consequence of the MEHHP exposure. These findings underscore the intricate interplay between phthalate exposure and the immune system in the context of organoid models.

Among the cytokines influenced by MEHHP exposure in MYOF organoids is IL‐13, an immune‐modulating cytokine that may influence various cellular processes within the MYOF organoids. Eotaxin, involved in recruiting immune cells [112], could indicate an inflammatory response to MEHHP exposure [78]. FGF‐2, a potent growth factor, might play a role in cellular proliferation and tissue repair following injury caused by phthalate exposure [113]. M‐CSF and G‐CSF are crucial for the differentiation and activation of macrophages and granulocytes, respectively, suggesting an immune response to the toxic insult. FLT‐3L likely influences the development of immune cells and hematopoiesis in the organoids [114]. EGF is known for its role in tissue regeneration and cell growth, and its altered levels could signify an attempt to repair MEHHP‐induced damage. GM‐CSF, essential for immune cell production, might also be involved in the immune response within the organoids. MCP‐1, an important chemokine, likely participates in recruiting monocytes to the site of exposure [115]. IL‐4 and IL‐10, both anti‐inflammatory cytokines, might indicate a regulatory response to counterbalance the inflammation triggered by MEHHP. GROα, involved in cell proliferation and migration, could influence tissue repair processes in response to phthalate exposure [116]. Finally, TGFβ1, a potent regulator of cell growth and differentiation, might play a role in orchestrating the cellular response to the toxic insult. Additionally, chronic inflammation within the uterine environment further augments UF proliferation through the release of pro‐inflammatory cytokines and chemokines, exacerbating angiogenesis and fibrotic processes [23, 24]. The interplay between OS and inflammation perpetuates fibroid growth, underlining the significance of this relationship in understanding UF pathobiology and shaping diagnostic and therapeutic implications.

Importantly, MEHHP exposure was associated with increased 8‐OHdG expression, a biomarker of oxidative DNA damage, suggesting that phthalate‐induced ROS may contribute to mutations in genes critical to UF development, including *MED12* [117, 118]. Although causality requires further investigation, this aligns with evidence linking phthalate exposure, OS, and *MED12* mutation‐driven tumorigenesis.

Mitochondrial DNA (mtDNA) alterations provide additional mechanistic insight. mtDNA copy number reflects OS and bioenergetic demand, and its dysregulation following MEHHP exposure supports mitochondrial involvement as an early event leading to ECM remodeling and inflammation [119, 120]. Collectively, these findings support a mechanistic model in which MEHHP exposure exacerbates mitochondrial dysfunction and OS in at‐risk MMSCs, driving downstream ECM accumulation, cytokine dysregulation, and fibroid phenotype emergence.

In summary, our results suggest that exposure to Phthalates induces elevated levels of TGF‐β1, which in turn can contribute to increased DNA damage and OS, ultimately culminating in *MED12* mutation [29, 118]. The study underscores the intricate interplay of inflammatory and immune‐related processes in MYOF organoids when exposed to MEHHP. However, it is important to acknowledge that further research is necessary to explore the long‐term effects of MEHHP exposure and its impact on fibroid development in vivo using EKER rat model for better visualization of myometrial immune cells' infiltration and tissue stiffness. Understanding the relationship between phthalate exposure and the pathogenesis of UF requires comprehensive investigations. The identification of specific cytokine groups associated with inflammation, immune response, and tissue remodeling lays a solid foundation for exploring targeted therapeutic strategies aimed at mitigating the impact of phthalate exposure on fibroid development and potentially improving clinical outcomes for affected individuals who are at higher risk of EDCs exposure and thus UF development.

Although increased proliferation may contribute to higher absolute cytokine and ECM levels, the much larger MEHHP‐induced fold changes compared with viability or apoptosis strongly support a direct biological effect of MEHHP on inflammatory and ECM pathways; we acknowledge that normalizing secreted marker levels to proliferation would provide additional precision. This constitutes a limitation of the current study, alongside the relatively small sample size and the use of an organoid‐based in vitro model rather than an in vivo system. Despite these limitations, our findings support that phthalate exposure, particularly MEHHP, can act as a secondary environmental hit, amplifying mitochondrial dysfunction and inflammatory pathways in predisposed MMSCs and thereby promoting UF pathogenesis. Importantly, identifying specific cytokine and ECM signatures associated with MEHHP exposure may inform the development of targeted preventive and therapeutic strategies for women at heightened risk of UF development.

## 5 Conclusion

This study demonstrates that phthalate exposure directly promotes a pro‐fibroid environment in primed MYOF organoids, characterized by significantly increased cell viability, reduced apoptosis, and specific extracellular matrix (ECM) remodeling. The underlying mechanisms involve OXPHOS‐related mitochondrial dysfunction and a distinct inflammatory cytokine profile. These findings underscore the critical role of environmental triggers in UF pathogenesis and reveal promising therapeutic targets in ECM modulation and mitochondrial function to mitigate UF development.

## Author Contributions

S.V. made the major contribution to the acquisition, analysis, and interpretation of the data, and drafted the manuscript. Conceptualization, A.A.‐H., M.A., Q.Y., A.R.Z.; Methodology, S.V., A.A.‐H., M.A., Q.Y.; Software and formal analysis, S.V., M.A.; Investigation, resources, and data curation, S.V., M.A., M.M.O.; Writing original draft preparation, S.V. and M.A.; Review and editing, S.V., M.A., A.A.‐H., M.M.O., A.R.Z., S.M.‐L., Q.Y.; Visualization, supervision, project administration, and funding acquisition, A.A.‐H, M.A. All authors have read and agreed to the published version of the manuscript.

## Funding

This study was partly supported by National Institutes of Health (NIH) grants (RO1 ES028615, RO1 HD094378, U54 MD007602, RO1 HD087417, and RO1 HD106285) (Ayman Al‐Hendy) as well as Society of Endometriosis and Uterine Disorders (SEUD) research grant (Mohamed Ali).

## Consent

Human tissue samples used in this study were obtained from patients who had previously provided informed consent for research use of surgical specimens through the University of Chicago tissue bank (IRB#20‐1414). This study involved secondary use of banked human tissue and did not include prospective enrollment or clinical trial participation.

## Conflicts of Interest

The authors declare that they have no competing interests. The National Institutes of Health provided Ayman Al‐Hendy reports. Ayman Al‐Hendy reports a relationship with Myovant Sciences Ltd. and Pfizer, including consulting or advisory. Also, Dr. Ayman Al‐Hendy is the founder of the INOFFA company.

## Supporting information


**Figure S1:** (A) MYOF and MYON stem cells‐derived 3D organoids. Representative images of the development of organoids from MMSCs. On day 0, ~1 × 104 MMSCs were mixed with 2 μL of Matrigel in 100 μL of media and grown in a V‐bottom 96‐well plate. On Day 7, each spheroid was treated with MEHHP 1.6 μM at 48 h. time points. (B) Differential expression gene clustering heatmap: This heatmap represents the results of FPKM cluster analysis, with genes clustered using the log_2_ (FPKM+1) values. Genes with high expression levels are shown in red, whereas those with low expression levels are depicted in green. The color gradient, ranging from red to green, signifies log_2_ (FPKM+1) values from large to small. Additionally, the heatmap includes information about each gene's chromosome, length, and biological type.
**Figure S2:** Bioinformatic analysis of MEHHP‐exposed MYOF group as compared to the MEHHP‐exposed MYON group. (A, B) MYOF‐MEHHP group and the MYON‐MEHHP group shared 9122 genes and 10 061 genes, respectively (term “shared” refers to the total number of common genes detected in all three technical replicates of the same treatment). (C) Among these genes, 3064 showed upregulation, indicating higher expression levels, whereas 2009 were downregulated, indicating lower expression levels (a positive log_2_FoldChange (log_2_FC) indicates higher expression in the MEHHP‐treated group, whereas a negative log_2_FC indicates lower expression in gene lists with a significance threshold of *p < 0.05). (D) GO enrichment analysis for BP, oxidative phosphorylation (OXPHOS) exhibited statistical significance. CC, the mitochondrial protein complex displayed significance, whereas NADH dehydrogenase activity was identified as an important MF. (E) In the KEGG pathway analysis, we observed a high expression of Oxidative phosphorylation (OXPHOS). (F) Pathways related to Respiratory electron transport, ATP synthesis by chemiosmotic coupling, and heat production by uncoupling proteins were notably significant in Reactome.
**Figure S3:** Bioinformatic analysis of MEHHP‐exposed MYOF group as compared to the VEH‐exposed MYOF group. (A, B) The Venn diagram showed an overlap of 9122 genes between these two groups, and the MYOF‐VEH group had an additional 8906 common genes in all three technical replicates of the same treatment. (C) Among these genes, 915 showed upregulation, and 652 displayed downregulation (a positive log_2_FoldChange (log_2_FC) indicates higher expression in the MEHHP‐treated group, whereas a negative log_2_FC indicates lower expression in gene lists with a significance threshold of *p < 0.05). (D) The intermediate filaments were particularly significant in CC based on GO enrichment analysis. (E) The KEGG pathway analysis revealed high expression levels in both Oxidative phosphorylation (OXPHOS) and Thermogenesis. (F) Reactome pathways also highlighted the significance of Respiratory electron transport, ATP synthesis by chemiosmotic coupling, and heat production by uncoupling proteins.
**Figure S4:** Bioinformatic analysis of MYON‐MEHHP group as compared to the MYON‐VEH group. (A, B) The MYON‐MEHHP group exhibited 10 061 genes in common with the MYON‐VEH group, whereas the latter group had 8997 unique genes. (C) Among these genes, 478 showed upregulation, and 826 displayed downregulation. (D) GO enrichment analysis indicated that BP related to ECM organization were statistically significant. Moreover, CC was enriched with extracellular matrix components, and receptor regulator activity played an important role in MF. (E) In the KEGG pathway analysis, Focal adhesion was highly expressed. (F) Reactome pathways highlighted the significance of ECM.
**Figure S5:** Bioinformatic analysis of MYOF‐VEH group as compared to the MYON‐VEH group. (A, B) These groups shared 8906 and 8997 genes, respectively. (C) Among these genes, 856 upregulated and 794 genes downregulated. (D) According to GO enrichment analysis, BP related to reproductive system development exhibited statistical significance. Additionally, the CC were significantly enriched with the proteinaceous ECM, and glycosaminoglycan played a vital role in MF. (E) KEGG analysis did not show any significant pathway. (F) Reactome pathways suggested higher expression levels in Interleukin‐4 and 13 signaling pathways.
**Figure S6:** Bioinformatic analysis showed oxidative phosphorylation (OXPHOS) imbalance in MYOF organoids Induced by MEHHP exposure (A, B, C) We identified 19 genes that were uniquely expressed in the MEHHP‐exposed MYOF group, distinguishing them from both the MEHHP‐exposed MYON and VEH‐exposed MYOF groups. (D) The results from the GSEA analysis highlighted the significance of oxidative phosphorylation (OXPHOS). (E) Based on these compelling findings, we carefully selected six up regulated genes for further investigation. Error bars represent the mean + s.d. *p < 0.05; **p < 0.01; ***p < 0.001. n.s., no significant difference. *p* values were analyzed by unpaired two‐tailed Student's *t*‐test and two‐way ANOVA.

**Table S1:** List of the 19 genes uniquely expressed in the MYOF‐MEHHP group, identified by intersecting differentially expressed genes between MYOF‐MEHHP versus MYON‐MEHHP and excluding genes that were differentially expressed in MYOF‐MEHHP versus MYOF‐VEH comparisons. These genes represent MYOF‐specific transcriptional responses to MEHHP exposure and were subsequently used for Gene Ontology (GO) and KEGG pathway enrichment analyses.

## Data Availability

Raw data was generated at the University of Chicago. Derived data supporting the findings of this study are available from the corresponding author on request. The data are not publicly available due to containing information that could compromise participants’ Ethical approval and consent to participate.
