# Supplementary material for: Phthalate Metabolite, Mono(2‐Ethyl‐5‐Hydroxyhexyl) Phthalate (MEHHP), Promotes Uterine‐Fibroid–Associated Phenotypes in Myometrial Stem Cell‐Derived 3D Organoids
Source: Environ Toxicol. 2026 Feb 1;41(10):975–97. doi: 10.1002/tox.70046 (PMC13537346; doi:10.1002/tox.70046)
Supplement: Supplementary file 1 [file TOX-41-975-s002.docx]

| **MYOF-MEHHP vs MYON-MEHHP**  **GO** | **MYOF-MEHHP vs MYOF-VEH** | **19 common elements** |
| --- | --- | --- |
| NDUFA4L2 | MGST2 | ATP5F1D |
| MT-CYB | NDUFA9 | ATP5MC1 |
| MT-ND3 | CYBA | ATP5PD |
| MT-CO1 | ABL2 | ATP5PO |
| MT-ND4L | AL031284.1 | COX6A1 |
| MT-ND2 | GSTM2 | COX6B1 |
| MT-ATP6 | GSTO1 | COX6C |
| MTND1P23 | FOS | COX7A1 |
| MT-ND6 | AL161645.1 | COX8A |
| MT-ND4 | CAT | NDUFA1 |
| MT-ND1 | COX7A1 | NDUFA11 |
| COX7A1 | VDAC1P8 | NDUFA3 |
| MT-CO2 | NDUFS5 | NDUFA9 |
| MT-ATP8 | NDUFA1 | NDUFB1 |
| MTND2P28 | NDUFB8 | NDUFB7 |
| MT-ND5 | MET | NDUFB8 |
| ATP5ME | RAC1 | NDUFB9 |
| MT-CO3 | NDUFA11 | NDUFS5 |
| NDUFA11 | COX6C | NDUFS6 |
| MTND4P12 | COX6B1 |  |
| COX6C | PIK3R1 |  |
| TCIRG1 | MAPK13 |  |
| COX8A | HRAS |  |
| MTCO3P12 | ATP5PO |  |
| NDUFA1 | NFE2L2 |  |
| ATP6V0C | MAP3K14 |  |
| MTATP6P1 | AC253536.2 |  |
| NDUFB8 | COX6A1 |  |
| NDUFA9 | ATP5PD |  |
| COX6B1 | NDUFB1 |  |
| COX6A1 | GSTT2B |  |
| ATP5PO | NDUFB9 |  |
| LHPP | NDUFS6 |  |
| NDUFS5 | COX8A |  |
| ATP5MC1 | NDUFB7 |  |
| NDUFB7 | NDUFA3 |  |
| ATP5PD | ATP5F1D |  |
| NDUFB1 | MTCO3P9 |  |
| ATP5F1D | MTATP6P24 |  |
| NDUFA3 | ATP5MC1 |  |
| NDUFB9 |  |  |
| ATP6V1F |  |  |
| ATP6V0E2 |  |  |
| NDUFA5 |  |  |
| UQCRQ |  |  |
| COX5B |  |  |
| NDUFB11 |  |  |
| MTCO1P12 |  |  |
| COX7B |  |  |
| COX5BP6 |  |  |
| ATP6V1C2 |  |  |
| NDUFB10 |  |  |
| COX6A1P2 |  |  |
| ATP5F1C |  |  |
| ATP5MG |  |  |
| COX4I1 |  |  |
| NDUFS8 |  |  |
| UQCR11 |  |  |
| NDUFV3 |  |  |
| UQCRB |  |  |
| UQCR10 |  |  |
| UQCRC2 |  |  |
| NDUFS3 |  |  |
| NDUFA6 |  |  |
| NDUFB4 |  |  |
| ATP6V1B2 |  |  |
| ATP5MFP4 |  |  |
| NDUFC2 |  |  |
| SDHA |  |  |
| NDUFA4 |  |  |
| VAX2 |  |  |
| UQCRC1 |  |  |
| NDUFS6 |  |  |
| ATP6V0B |  |  |

| **MYOF-MEHHP vs MYON-MEHHP**  **KEGG** | **MYOF-MEHHP vs MYOF-VEH** | **19 common elements** |
| --- | --- | --- |
| MTND1P23 | FOS | COX7A1 |
| MT-ND1 | COX7A1 | NDUFA11 |
| MT-ATP6 | GSTO1 | COX6C |
| MT-ND6 | AL161645.1 | COX8A |
| MT-ND4 | CAT | NDUFA1 |
| MT-ND5 | MET | NDUFB8 |
| MT-CO2 | NDUFS5 | NDUFA9 |
| MT-ND2 | GSTM2 | COX6B1 |
| MT-ND4L | AL031284.1 | COX6A1 |
| MT-CO1 | ABL2 | ATP5PO |
| MT-CO3 | NDUFA11 | NDUFS5 |
| MT-CYB | NDUFA9 | ATP5MC1 |
| MTND2P28 | NDUFB8 | NDUFB7 |
| MT-ND3 | CYBA | ATP5PD |
| MT-ATP8 | NDUFA1 | NDUFB1 |
| NDUFA4L2 | MGST2 | ATP5F1D |
| COX7A1 | VDAC1P8 | NDUFA3 |
| ATP5ME | RAC1 | NDUFB9 |
| NDUFA11 | COX6C | NDUFS6 |
| MTND4P12 | COX6B1 |  |
| COX6C | PIK3R1 |  |
| TCIRG1 | MAPK13 |  |
| COX8A | HRAS |  |
| MTCO3P12 | ATP5PO |  |
| NDUFA1 | NFE2L2 |  |
| ATP6V0C | MAP3K14 |  |
| MTATP6P1 | AC253536.2 |  |
| NDUFB8 | COX6A1 |  |
| NDUFA9 | ATP5PD |  |
| COX6B1 | NDUFB1 |  |
| COX6A1 | GSTT2B |  |
| ATP5PO | NDUFB9 |  |
| LHPP | NDUFS6 |  |
| NDUFS5 | COX8A |  |
| ATP5MC1 | NDUFB7 |  |
| NDUFB7 | NDUFA3 |  |
| ATP5PD | ATP5F1D |  |
| NDUFB1 | MTCO3P9 |  |
| ATP5F1D | MTATP6P24 |  |
| NDUFA3 | ATP5MC1 |  |
| NDUFB9 |  |  |
| ATP6V1F |  |  |
| ATP6V0E2 |  |  |
| NDUFA5 |  |  |
| UQCRQ |  |  |
| COX5B |  |  |
| NDUFB11 |  |  |
| MTCO1P12 |  |  |
| COX7B |  |  |
| COX5BP6 |  |  |
| ATP6V1C2 |  |  |
| NDUFB10 |  |  |
| COX6A1P2 |  |  |
| ATP5F1C |  |  |
| ATP5MG |  |  |
| COX4I1 |  |  |
| NDUFS8 |  |  |
| UQCR11 |  |  |
| NDUFV3 |  |  |
| UQCRB |  |  |
| UQCR10 |  |  |
| UQCRC2 |  |  |
| NDUFS3 |  |  |
| NDUFA6 |  |  |
| NDUFB4 |  |  |
| ATP6V1B2 |  |  |
| ATP5MFP4 |  |  |
| NDUFC2 |  |  |
| SDHA |  |  |
| NDUFA4 |  |  |
| VAX2 |  |  |
| UQCRC1 |  |  |
| NDUFS6 |  |  |
| ATP6V0B |  |  |
